# Alkali Metals Activated High Entropy Double Perovskites for Boosted Hydrogen Evolution Reaction

**DOI:** 10.1002/advs.202406453

**Published:** 2024-09-09

**Authors:** Ning Sun, Zhuangzhuang Lai, Wenbo Ding, Wenbo Li, Tianyi Wang, Zhichuan Zheng, Bowen Zhang, Xiangjiang Dong, Peng Wei, Peng Du, Zhiwei Hu, Chih‐Wen Pao, Wei‐Hsiang Huang, Haifeng Wang, Ming Lei, Kai Huang, Runze Yu

**Affiliations:** ^1^ State Key Laboratory of Information Photonics and Optical Communications School of Science Beijing University of Posts and Telecommunications Beijing 100876 P. R. China; ^2^ Center for High Pressure Science and Technology Advanced Research Beijing 100193 P. R. China; ^3^ State Key Laboratory for Green Chemistry Engineering and Industrial Catalysis Centre for Computational Chemistry and Research Institute of Industrial Catalysis School of Chemistry and Molecular Engineering East China University of Science and Technology Shanghai 200237 P. R. China; ^4^ Max Planck Institute for Chemical Physics of Solids Nothnitzer Strasse 40 01187 Dresden Germany; ^5^ National Synchrotron Radiation Research Center 101 Hsin‐Ann Road Hsinchu 300092 Taiwan

**Keywords:** alkali metal, double perovskite, high entropy, hydrogen evolution reaction, super‐exchange interaction

## Abstract

An efficient and facile water dissociation process plays a crucial role in enhancing the activity of alkaline hydrogen evolution reaction (HER). Considering the intricate influence between interfacial water and intermediates in typical catalytic systems, meticulously engineered catalysts should be developed by modulating electron configurations and optimizing surface chemical bonds. Here, a high‐entropy double perovskite (HEDP) electrocatalyst La_2_(Co_1/6_Ni_1/6_Mg_1/6_Zn_1/6_Na_1/6_Li_1/6_)RuO_6_, achieving a reduced overpotential of 40.7 mV at 10 mA cm^−2^ and maintaining exemplary stability over 82 h in a 1 m KOH electrolyte is reported. Advanced spectral characterization and first‐principles calculations elucidate the electron transfer from Ru to Co and Ni positions, facilitated by alkali metal‐induced super‐exchange interaction in high‐entropy crystals. This significantly optimizes hydrogen adsorption energy and lowers the water decomposition barrier. Concurrently, the super‐exchange interaction enhances orbital hybridization and narrows the bandgap, thus improving catalytic efficiency and adsorption capacity while mitigating hysteresis‐driven proton transfer. The high‐entropy framework also ensures structural stability and longevity in alkaline environments. The work provides further insights into the formation mechanisms of HEDP and offers guidelines for discovering advanced, efficient hydrogen evolution catalysts through super‐exchange interaction.

## Graphical abstract

Inspired by the fascinating “super‐exchange interaction,” a nanostructure high entropy double perovskite (HEDP) platform is developed to accelerate the hydrogen evolution reaction (HER) process, where the adsorption of intermediates can be enhanced owing to the alkali metal (AM^+^) induced orbital hybridization and electron reflux. The orbital hybridization optimizes the electron configuration of the active sites, providing new impetus for advances in renewable energy.

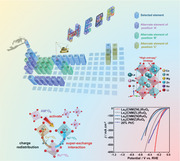

## 1 Introduction

Hydrogen has increasingly been recognized as a potent energy carrier and a viable alternative to fossil fuels, particularly under the prevailing theme of green, low‐carbon, and energy‐efficient development.^[^ ^1^, ^2^ ^]^ The International Energy Agency's analysis of global final energy consumption indicates that the proportion of hydrogen is projected to increase to ≈2% by 2030 and 10% by 2050, highlighting the pressing requirement for a green hydrogen strategy to fulfill the objectives of the net‐zero plan.^[^ ^3^, ^4^ ^]^ The alkaline hydrogen evolution reaction (HER) process can effectively mitigate concerns related to acidic corrosion and dissolution of active substances in electrocatalytic water splitting, producing high‐purity hydrogen gas (>99.7%) for diverse industrial applications.^[^ ^5^ ^]^ However, the kinetics of HER in alkaline conditions are significantly slower than in acidic media, due to the additional energy barrier for breaking the O─H bond within water dissociation in the Volmer step.^[^ ^6^, ^7^ ^]^ The chemistry of the interfacial water layer and pivotal intermediates profoundly determines the actual electrochemical performance.^[^ ^8^, ^9^, ^10^ ^]^ Therefore, it is imperative to elucidate the intrinsic relevance of the chemical state of active centers, establishing the connection between catalytic activity and potential mechanisms during HER.^[^ ^11^, ^12^, ^13^ ^]^ Much research has focused on regulating initial water adsorption, subsequent dissociation, hydroxyl affinity to the catalyst surface, and hydrogen intermediate adsorption by designing novel catalysts to facilitate rapid and efficient hydrogen evolution in alkaline media.^[^ ^14^, ^15^, ^16^ ^]^ However, the real catalytic centers and corresponding reaction pathways remain unclear due to the elusive interfacial coupling of active sites to intermediates with dynamically changing catalyst surfaces (e.g., amorphization and leaching of elements).^[^ ^17^ ^]^ Consequently, it is crucial to exploit an innovative electrocatalytic platform with a more stable structure to clarify the adsorption mechanism in the alkaline HER.

The pursuit of identifying appropriate additives to modulate the reaction pathway has garnered considerable attention as a promising approach. Currently, the introduction of alkali metal cations (AM^+^) plays a pivotal role in both laboratory and industrial catalytic fields.^[^ ^18^, ^19^, ^20^ ^]^ Through mechanisms such as doping, intercalation, coordination, and the use of alkaline electrolyte media, AM^+^ can promote the catalytic stability of materials by preventing structural collapse or agglomeration to a certain extent.^[^ ^21^, ^22^ ^]^ Additionally, the chemisorption of reaction intermediates, enhanced by AM^+^ through modulation of crystal structure and electron flow, is critical for improving water electrolysis. For instance, Na doping in SrRuO_3_ weakens Ru‐adsorbate intermediates and shifts the O *p*‐band and Ru *d*‐band centers positively, thus enhancing both activity and durability by optimizing intermediates adsorption.^[^ ^23^ ^]^ Similarly, smaller cations (Li^+^) favor higher OH_ad_ coverage, which promotes water dissociation and Volmer‐step kinetics on the Pt surface, thereby enhancing HER.^[^ ^24^ ^]^ Despite significant progress in the development of electrocatalysts, the contribution of AM^+^ to the electronic local environment of catalysts remains limited due to the constraints on doping quantities and the differences in electronegativity, valence, and ionic radius between AM^+^ and active elements. It's challenging for AM^+^ to provide electrons or act as electron acceptors directly for the active elements of electrocatalysts due to their limited orbital capacities and simple charge environments. Additionally, AM^+^ tend to dissolve or shed from intercalation and other locations at high potentials during electrochemical tuning, leading to potential structural instability. Furthermore, the existence of complexes ((H_2_O)_x_AM^+^) formed by AM‐OH strength and hydrogen bonding between hydration water and adsorbed OH_ad_ renders the mechanism of hydroxyl adsorbate coverage at electrocatalyst's active sites in alkaline solution elusive.^[^ ^25^, ^26^ ^]^ Therefore, elucidating the effect of these “chemically inert spectators” AM^+^ on the crystal platform for catalytic reactions and providing complex orbitals to regulate electronic behavior with AM^+^ have become imperative.^[^ ^27^ ^]^

Fortunately, entropy‐driven strategies possess the potential to address these limitations and amplify the advantages associated with AM^+^. High‐entropy materials (HEM) can combine five or more elements to create a stable solid–solution platform, thus granting virtually infinite degrees of freedom for manipulating atomic structure and composition.^[^ ^28^ ^]^ The notable diversity in composition and structure facilitates precise adjustments to the electronic structure of surface sites. This offers a promising opportunity for customizing the functional attributes of AM^+^ additives to optimize the adsorption capacity of reaction intermediates due to the highly disordered crystal structure, complex energy band environment, and overlapping multi‐orbital distribution of HEM.^[^ ^29^ ^]^ Recently, a variety of special AM^+^ additives through high entropy engineering have been explored in electrolytic water catalysts, cathode materials, solar cells, and solid‐state electrolytes.^[^ ^30^, ^31^, ^32^, ^33^, ^34^ ^]^ The intricate entropy stabilization mechanism ensures the unity of structural stability within the thermodynamic landscape and cycling endurance in industrial applications with AM^+^ additives. However, previous reports with AM^+^ introduced have predominantly modulated catalytic performance through vacancies and defects engineering strategies (owing to the vastly different ionic radii and electronegativities), neglecting the optimization of key intermediate adsorption through orbital and charge modification at active sites. Moreover, the mechanism of HER catalytic intermediate adsorption in high‐entropy systems via the introduction of AM^+^ to promote electron reflux or orbital hybridization remains unclear.

Here, we employ a solid‐state and ball‐milling strategy to fabricate a series of pure‐phase high entropy double perovskites (HEDP) electrocatalysts for alkaline HER. The optimized catalyst La_2_(CNMZNL)RuO_6_ demonstrates remarkable activity for HER in alkaline media, with a minimal overpotential of merely 40.7 mV at 10 mA cm^−2^, alongside robust stability for up to 82 h at 10 mA cm^−2^ and 24 h at 200 mA cm^−2^. Corresponding characterizations and density functional theory (DFT) analyses confirm that the AM^+^‐induced super‐exchange interaction promotes charge hopping between metal–oxygen–metal polyhedrons, consequently inducing multi‐orbital hybridization. This consequent unconventional charge redistribution optimizes the surface adsorption capacity of active sites on intermediates and reduces the potential barrier of water dissociation, thereby further enhancing the efficiency of alkaline HER. This study offers an opportunity to definitively elucidate the mechanism of alkaline HER via super‐exchange interaction and establishes a novel platform for HEDP electrocatalysts aimed at various heterogeneous catalytic processes.

## 2 Results and Discussion

### 2.1 Synthesis and Structural Characterization

As a typical crystal structure of B‐site ordered double perovskites, the fundamental composition of its chemical formula is denoted by A_2_B'B″O_6_. To assess the feasibility of high entropy incorporation of metal elements in the B′ position, a series of Ru‐based double perovskite materials were prepared by conventional solid‐state synthesis and high‐energy ball milling techniques (Figures S1 and S2, Supporting Information). Subsequently, a high‐entropy ruthenium‐based double perovskite material, incorporating AM^+^ elements, specifically La_2_Co_1/6_Ni_1/6_Mg_1/6_Zn_1/6_Na_1/6_Li_1/6_RuO_6_, was successfully synthesized (Figure S1, Supporting Information). Despite conventional lanthanide‐based catalysts posing a significant bottleneck in the HER reaction due to their poor conductivity and formidable water dissociation barrier, La‐based perovskite emerges as a novel rare‐earth‐based material. It features a distinct 4*f* orbital occupancy that profoundly influences electronic modulation, thereby enhancing electrocatalytic activity.^[^ ^35^ ^]^ To elucidate the influence of AM^+^ in ruthenium‐based double perovskite materials, electrocatalysts with varying configuration entropy within the same system were also prepared for comparative analysis. Furthermore, ten elements were selected for the crystal construction under high entropy strategy by screening the electronegativity, ionic radius, perovskite tolerance factor, and other relevant parameters of the candidate materials, confirming the universality of the preparation method (Figure S3, Supporting Information). This approach incorporates multiple elements with closely matched ionic radii and stable divalent states to enable the implementation of the high entropy strategy in the B′ position, alongside ruthenium elements possessing large ionic radii and high electronegativity in the B″ position. For simplicity, we have abbreviated the characterization of certain materials, such as La_2_(CNMZNL)RuO_6_ for the aforementioned target samples, with the remaining materials delineated in Table S1 (Supporting Information).

**Figure 1:**
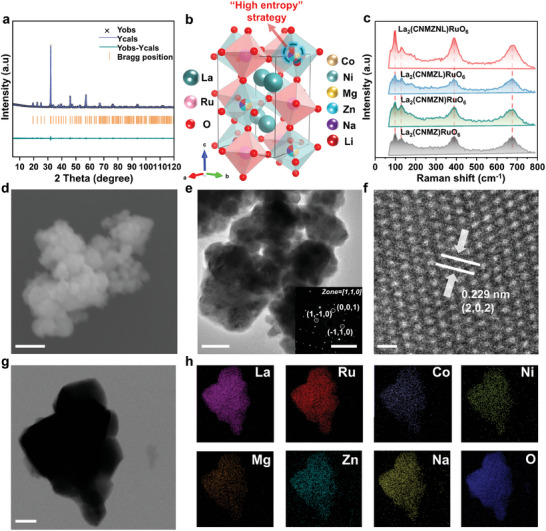
Characterization of La_2_(CNMZNL)RuO_6_ catalysts. a) XRD refinement. b) Schematic diagram of crystal structure. The dotted line represents the site of high entropy, which is proved by refinement and later characterization. c) Raman spectrum. d) SEM image. (scale bar: 50 nm). e) TEM image (scale bar: 50 nm) and the insert graph is SAED (scale bar: 5 1/nm). f) HR‐TEM image. (scale bar: 0.5 nm). g,h) EDS‐mapping image. (scale bar: 50 nm).

The powder X‐ray diffraction (XRD) patterns and Rietveld refined XRD patterns depicted in **Figure** **1**a and Figures S4 and S5 (Supporting Information), demonstrate that all Ru‐based double perovskites exhibit a single phase, crystallizing in a monoclinic structure with a space group of *P*2_1_/n.^[^ ^36^ ^]^ The principal peak clearly shifts due to variations in the radius of the doped elemental ions and the entropy‐driven structural strain. High‐entropy samples containing mixed AM^+^ ions, such as La_2_(CNMZNL)RuO_6_ and La_2_(CNMZNL)RuO_6_, exhibit heightened peak intensities ≈32.2°, suggesting reduced lattice strain.^[^ ^37^ ^]^ The unit cell volumes exhibit a gradual expansion with the increase of configuration entropy (Figure S5f, Supporting Information), attributed to the expansion effect of the high entropy and the alteration in ionic radius of the doped elements. It is noteworthy that upon the introduction of Li^+^, the factor contributing to the increase in crystal cell volume due to configuration entropy disappears, resulting in the diminishment of the cell owing to the smaller radius of Li^+^. The visualization model of high entropy double perovskite is established in Figure 1b.

Importantly, the successful preparation of B‐site ordered La_2_LiRuO_6_ crystal (Figure S1, Supporting Information) confirms that the AM^+^ including Na^+^ and Li^+^, preferentially occupy the B' site in La_2_B'RuO_6_. This results in a spatially ordered arrangement of Na/Li‐O_6_ octahedra and Ru–O_6_ octahedra, as supported by existing literature,^[^ ^38^, ^39^ ^]^ instead of replacing La sites or filling the lattice gaps. When the high entropy region occurs in the B' position, corner‐shared high‐entropy octahedron (HEO_6_) and interconnected RuO_6_ form columns along the “*c*” axis, with La cations positioned at the void positions between these octahedra. Furthermore, subsequent XRD refinement results indicate that AM^+^ exhibits a heightened propensity for occupying high‐entropy‐driven B' site, rather than snatching the La and Ru sites (Figure S6, Supporting Information). The tilting of Jahn–Teller distorted HEO_6_ and RuO_6_ octahedra is visible in the crystal structure, potentially enhancing the feasibility of electron transitions and orbital coupling (Table S2, Supporting Information). Raman spectroscopy as an effective tool has been used to further determine the crystal structure and molecular vibration. According to the group theory analysis, 24 Raman active modes can be expected for the samples with *P*2_1_/n symmetry. However, we just observe four relatively strong peaks at 683.78, 387.85, 132.77, and 99.73 cm^−1^ due to degeneracy and peak overlap of different frequency bands in Figure 1c. It is noteworthy that these results are in good agreement with those presented in the literature,^[^ ^40^ ^]^ thus corroborating the effectiveness of the high entropy strategy. Compared with other materials, the Raman peak position of La_2_(CNMZNL)RuO_6_ exhibits minimal variation, while the intensity of the peaks increases with the rise of configuration entropy factor, substantiating the high entropy nature and the alteration of microstructure in molecular vibration upon the introduction of AM^+^.^[^ ^41^ ^]^

The morphologies of La_2_(CNMZNL)RuO_6_ are depicted in Figure 1d,e. Scanning electron microscopy (SEM) images show that the material after ball milling has nano‐sized, ball‐like particles with a dominant size distribution of 20–50 nm (Figure 1d), significantly smaller than those before ball milling (Figure S7, Supporting Information). Transmission electron microscopy (TEM) suggests a nanostructure with a similar particle size (Figure 1e). The selected area electron diffraction (SAED) pattern further confirms that La_2_(CNMZNL)RuO_6_ presents a single‐crystalline feature, and the indexing data is consistent with the XRD data. High‐resolution TEM (HR‐TEM) image shows a lattice spacing of 0.229 nm (Figure 1f), which corresponds to the (202) crystal planes of the monoclinic perovskite oxide phase. The scanning transmission electron microscopy (STEM) mappings and SEM energy dispersion spectra (EDS) mapping also demonstrate the uniform distribution of all elements in the sample, nearly matching the nominal ratio (Figure 1g,h; Figure S8, Supporting Information). The elemental concentrations of the samples were determined using inductively coupled plasma optical emission spectrometer (ICP‐OES) analysis (Table S3, Supporting Information). The composition of the high entropy site aligns with the Co site composition in the parent compound La_2_CoRuO_6_, demonstrating a nearly proportional distribution of the metallic elements Co, Ni, Mg, Zn, Na, and Li. However, the concentration of Na is slightly lower compared to other metallic elements occupying the same Wyckoff position, attributed to partial evaporation at higher temperatures and competitive interactions of AM^+^ within the lattice.^[^ ^23^, ^42^ ^]^ Moreover, Li^+^ may be more likely to occupy high entropy sites due to its closer ionic radius (76 pm) and electronegativity relative to the transition metal elements. This gives them a competitive advantage position over Na^+^ (102 pm) for elemental ratios.^[^ ^33^ ^]^ To keep the pure phase, the proportion of AM^+^ in the high entropy framework cannot be increased indefinitely (Figure S9, Supporting Information). Therefore, we believe that the HEPD of La_2_(CNMZNL)RuO_6_ with pure‐phase structure, nano‐spherical morphology, and introduction of AM^+^ has been formed. The nanostructure may provide a potentially high specific surface area, while the high‐entropy characteristics and Jahn–Teller‐distorted HEO_6_ octahedra provide a framework for customized orbital electron design in the subsequent discussion.

### 2.2 Charge Redistribution via Super‐Exchange Interaction

**Figure 2:**
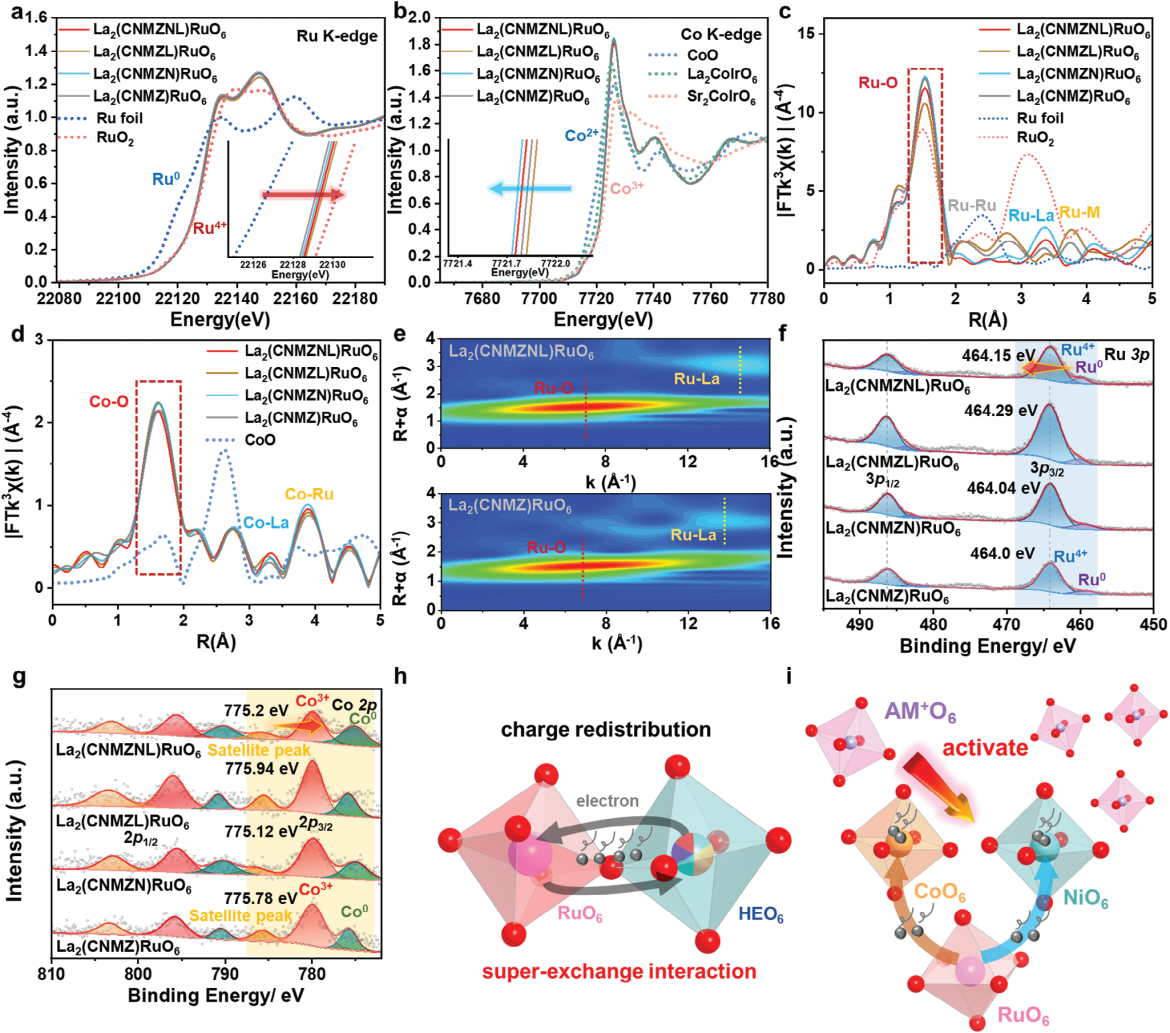
The electronic structure and local coordination environment with AM^+^ for La_2_(CNMZN)RuO_6_. XANES spectra of a) Ru K‐edge, b) Co K‐edge. EXAFS spectra of c) Ru, d) Co. WT‐EXAFS images of Ru of e) for La_2_(CNMZNL)RuO_6_ and La_2_(CNMZ)RuO_6_. High‐resolution of XPS spectra of f) Ru 3*p* and g) Co 2*p*. h,i) Schematic illustration of the super‐exchange interaction and charge redistribution with AM^+^ in La_2_(CNMZNL)RuO_6_.

To elucidate the impact of the AM^+^ additive and the corresponding super‐exchange interaction mechanism in HEDP catalysts, synchrotron‐based X‐ray absorption spectroscopy (XAS) measurements were conducted to probe the electronic structure information. X‐ray absorption near‐edge structure (XANES) and extended X‐ray absorption fine structure (EXAFS) were employed to investigate the valence states and local atomic configurations of Ru, Co, Ni, and Zn within the selected catalysts La_2_(CNMZNL)RuO_6_. As depicted in **Figure** **2**a, the rise in the absorption edge implies an elevation in the Ru valence upon the inclusion of the Na^+^ and Li^+^ elements in La_2_(CNMZNL)RuO_6_, compared with La_2_(CNMZ)RuO_6_ alternative. Specifically, the valence states of Ru within the samples are estimated to be +3.77 (La_2_(CNMZNL)RuO_6_) and +3.61 (La_2_(CNMZ)RuO_6_), with references to Ru(0) foil and Ru(+4)O_2_ (Figure S10a, Supporting Information). However, both the normalized absorption position of Co and Ni *K*‐edges in La_2_(CNMZNL)RuO_6_ exhibit a slight leftward left, indicating electron transfer from neighboring atoms to both Co and Ni atoms and the corresponding valence changes (Figure 2b; Figure S10b–d, Supporting Information).^[^ ^43^, ^44^ ^]^ Furthermore, the marginal rightward shift of the Zn *K*‐edge in comparison to La_2_(CNMZ)RuO_6_ indicates the normalized absorption position of the Zn element to donate electrons toward Co, Ni, etc. (Figure S8e, Supporting Information). Based on the above XANES study, La_2_(CNMZNL)RuO_6_ possess a moderate Ru valence (Ru^3.77+^), Co valence (Co^2.50+^), and Ni valence (Ni^+2.18^), while the ions of La^3+^, Zn^2+^, Na^+^, Li^+^ are more likely to remain in their original normal valence states due to their inherent inertia. Since there are few oxygen vacancies to observe (see below), the most probable scenario for maintaining charge equilibrium is that the lattice oxygen (O^(2−n)−^) in super‐exchange interactions provides additional electrons and undergoes slight oxidation. Furthermore, the system's charge can be equilibrated through the reorganization of elemental electrons, as demonstrated by DFT analysis (Table S4, Supporting Information). The results described above collectively suggest the redistribution of electrons and the precise adjustment of the electronic structure in La_2_(CNMZNL)RuO_6._ Despite the higher electronegativity of Ru (2.20) compared to both Co (1.88) and Ni (1.91) in La_2_(CNMZNL)RuO_6_, the introduction of AM^+^ species facilitates electron transfer from Ru to Co and Ni, consequently leading to anomalous charge rearrangement.

The EXAFS spectra were utilized to further determine the coordination structure of the four absorbing metallic elements in the La_2_(CNMZNL)RuO_6_ in Figure 2c,d, and Figure S10 (Supporting Information). Fourier transforms (FT; No phase correction) of these EXAFS spectra, shown as experimental k^2^‐weighted EXAFS oscillations,^[^ ^45^, ^46^, ^47^, ^48^ ^]^ provide detailed insights into the local atomic structures of elements within the La_2_(CNMZNL)RuO_6_ catalysts (Table S5, Supporting Information). The La_2_(CNMZNL)RuO_6_ sample distinctly exhibits a peak at 2.01 Å corresponding to the Ru─O scattering path. Additional peaks at ≈3.49 and 3.96 Å are observed, indicating the Ru–La coordination (Figure 2c; Table S5, Supporting Information). A peak at 4.07 Å corresponds to the Ru─M scattering path in La_2_(CNMZNL)RuO_6_, indicating the average distance between Ru in the B' site and the high‐entropy metal elements in the B' sites. Notably, there is no characteristic peak for Ru–Ru scattering at 2.45 Å (phase uncorrected distance) in La_2_(CNMZNL)RuO_6_, which is present in Ru foil and RuO_2_. In the EXAFS spectra presented in Figure 2d, La_2_(CNMZNL)RuO_6_ exhibits a first shell Co–O peak at 2.01 Å, with additional peaks at 3.11  and 3.31 Å corresponding to Co–La interactions, and a Co─Ru peak at 3.95 Å in the higher shell. Moreover, the Co–Co metal shell (at ≈2.18 Å, as the previous literature reported) is absent in the target sample La_2_(CNMZNL)RuO_6_ indicating no Co exsolution from the lattice.^[^ ^49^ ^]^ The EXAFS spectra for Ni and Zn display similar coordination environments to Co (Figure S11, Supporting Information). All these FT curves for the first shell exhibit nearly identical distances and amplitudes for the Co, Ni, and Zn absorbers, despite having sites different from Ru (Figures S12 and S13, Supporting Information). This suggests that these high‐entropy metal cations possess an identical local atomic structure and are uniformly distributed in a completely random manner in the B' site within La_2_(CNMZNL)RuO_6_, without occupying the Ru sites. Consequently, HEO_6_ and RuO_6_ octahedrons are orderly arranged in the double perovskite, consistent with XRD refinement results. The fitting results suggest that the coordination numbers (CNs) of the shells show some change for all probed metals after the high‐entropy strategy. The wavelet transform (WT)‐EXAFS analysis reveals the first shell of La_2_(CNMZNL)RuO_6_ domain at R = 1.52 Å and k = 6.98 Å^−1^ is similar to La_2_(CNMZ)RuO_6_ (R = 1.52 Å and k = 7.03 Å^−1^) in Figure 2e. Conversely, the second shell layer attributed to the La─O scattering path illustrates that La_2_(CNMZ)RuO_6_ compound is distinguished from La_2_(CNMZNL)RuO_6_ due to the partial variations in local coordination environments introduced by AM^+^ elements. The WT‐EXAFS images for Co, Ni, and Zn in La_2_(CNMZNL)RuO_6_ are depicted in Figures S14 and S16 (Supporting Information), and exhibit similar coordination environments. These XAS results illustrate the electron interaction between HEO_6_ and RuO_6_ octahedra, including electron transfer from Ru to Co and Ni species in the La_2_(CNMZNL)RuO_6_. The incorporation of AM^+^ facilitates super‐exchange interaction, allowing electrons to transfer between the octahedra via oxygen‐ion bridges, potentially optimizing the adsorption capacity for reaction intermediates. Furthermore, the substantial disparity in ionic radii not only induces alterations in the local coordination environment but also stimulates orbital hybridization and rearrangement of charge density at the Fermi level, thereby activating the super‐exchange interaction in HEDP.

To deepen our comprehension of the underlying super‐exchange interaction mechanism that governs charge distribution and orbital hybridization of multi‐elements, we performed X‐ray photoelectron spectroscopy (XPS) to investigate surface chemistry. The XPS results are presented in Figure 2f,g and Figures S17 and S18 (Supporting Information). To specifically investigate the influence of AM^+^ elements on the surface valence state, we used La_2_(CNMZ)RuO_6_, La_2_(CNMZN)RuO_6_, and La_2_(CNMZL)RuO_6_ samples as references within HEDP. The XPS survey spectra confirm the presence of La, Co, Ni, Mg, Zn, Na, Li, Ru, and O on the La_2_(CNMZNL)RuO_6_ surface (Figure S17a, Supporting Information). For the ruthenium element, the surface is predominantly characterized by Ru^4+^ 3*p*_2/3_ (464.15 eV), but also contains some metallic ruthenium Ru^0^ 3*p*_2/3_ at 460 eV, as shown in Figure 2f. The binding energy of Ru^4+^ in La_2_(CNMZNL)RuO_6_ shifts toward higher energy by 0.15 eV compared to that in La_2_(CNMZ)RuO_6_.^[^ ^50^ ^]^ This indicates that Ru as the major active sites possess higher potential energy, which may prevent Ru^4+^ dissolution and Ru^0^ precipitation during the reduction reaction in HER. For the cobalt component, the XPS spectra of La_2_(CNMZNL)RuO_6_ display three 2*p*_3/2_ peaks: metal Co^0^ 2*p*_3/2_ (775.2 eV), Co^3+^ 2*p*_3/2_ (780.05 eV), and a satellite peak Co 2*p*_3/2_, as shown in Figure 2g.^[^ ^51^, ^52^ ^]^ Additionally, the XPS spectra confirm a decreased valence state of cobalt, evidenced by the negative shift of the binding peak by ≈0.58 eV following the introduction of Na^+^ and Li^+^. The Co^0^/Co^3+^ content changes from 51% for La_2_(CNMZ)RuO_6_ to 73% for La_2_(CNMZNL)RuO_6_, indicating a decrease in the average valence of cobalt. Similarly, the Ni 3*p* XPS spectra exhibit two binding peaks of Ni^2+^ 3*p*_3/2_ and Ni^2+^ 3*p*_1/2_ located at ≈66.89 and 68.45 eV, respectively (Figure S17b, Supporting Information).^[^ ^53^ ^]^ The binding energy of Ni 3*p*_2/3_ shifts negatively by 0.51 eV in the La_2_(CNMZNL)RuO_6_ sample compared to the middle entropy sample. These findings indicate that the inclusion of AM^+^ triggers a state of electron deficiency in ruthenium while fostering electron‐rich states in nickel and cobalt.

Lanthanides typically make minimal contributions to the electronic structure. However, after the introduction of the AM^+^, the binding energy of La in La_2_(CNMZNL)RuO_6_ also shifts toward lower binding energies compared to La_2_(CNMZ)RuO_6_, indicating its role as an electron reservoir. The existence of a doublet splitting of the La 3*d*_5/2_ peaks is consistent with reported literature values, appearing at 834.13 and 837.92 eV (Figure S17c, Supporting Information). For the oxygen element, the O 1*s* peak can be decomposed into three peaks corresponding to surface‐adsorbed water (≈532.63 eV), O^(2−n)−^ species with low electron density (≈531.27 eV), and lattice oxygen O^2−^ (≈529.12 eV), respectively (Figure S17d, Supporting Information).^[^ ^54^, ^55^, ^56^, ^57^ ^]^ Remarkably, the ratio of O^(2−n)−^ to O^2−^ in La_2_(CNMZNL)RuO_6_ is significantly higher than in La_2_(CNMZ)RuO_6_, highlighting oxygen's role as an electron donor and its involvement in electron flow through the octahedral bridge. The magnesium XPS spectra show that Mg primarily exists in the form of Mg^2^⁺, with a small proportion in the metallic state, at 1303.07 and 1304.50 eV respectively, with almost no shift in binding energy (Figure S18a, Supporting Information). In contrast, the Zn XPS spectrum presents only one pair of spin–orbit doublets at 1020.95 eV (2*p*_3/2_) and 1044.08 eV (2*p*_1/2_) without shakeup satellite, attributed to Zn^2+^ (Figure S16b, Supporting Information). Regarding the effect of AM^+^ elements, the characteristic peaks of Na 1*s* and Li 1*s* are resolved at 1071.46 and 54.25 eV, respectively (Figure S16c,d, Supporting Information). With the addition of AM^+^ elements, the binding energy of Ru and Zn increases, while the binding energy of La, Co, Ni, and O decreases in La_2_(CNMZNL)RuO_6_. This is similar to the XAS results, indicating that the super‐exchange interaction involves nearly all constituent elements in the high‐entropy system in electron redistribution (Figure 2h). However, there exists a distinction between the surface and bulk states within the crystal framework, characterized by unusual metallic states and greater electron accumulation observed at the surface. This is primarily due to the overlap of redox potentials influenced by the mechanism of the super‐exchange interactions, which accumulate electrons on the sample surface, thereby reducing the valence state of surface metals.^[^ ^58^ ^]^ Intriguingly, AM^+^ activates the complex super‐exchange interaction, optimizing the electronic configuration of the active elements (Figure 2i).

### 2.3 Energy Band Representation and Orbital Hybridization

**Figure 3:**
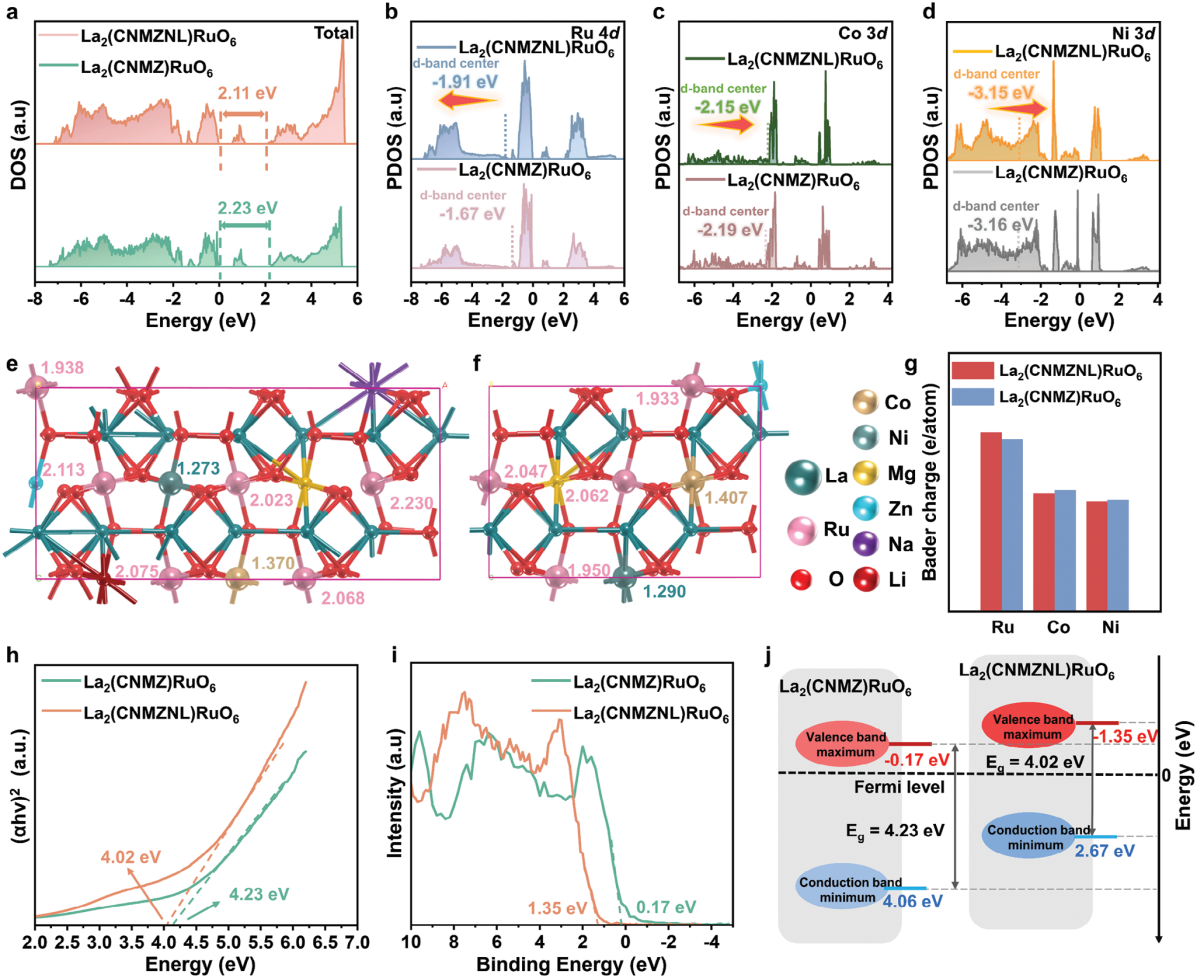
Energy band structure and corresponding theoretical calculations. a) Total DOS of La_2_(CNMZNL)RuO_6_ and La_2_(CNMZ)RuO_6_. The projected DOS (PDOS) of b) Ru 4*d*, c) Co 3*d*, and d) Ni 3*d*. e–g) Bader analysis of key elements (Ru, Co, and Ni) in La_2_(CNMZNL)RuO_6_ and La_2_(CNMZ)RuO_6_. h) Tauc plots of UV–vis spectroscopy for La_2_(CNMZNL)RuO_6_ and La_2_(CNMZ)RuO_6_. i) VB‐XPS spectrum La_2_(CNMZNL)RuO_6_ and La_2_(CNMZ)RuO_6_. j) Schematic of energy bands.

The systematic DFT calculations confirm the influence of AM^+^ doping on the electronic properties of the HEDP materials. Compared with La_2_(CNMZ)RuO_6_, the enhanced orbital extension and alignment of the *p*‐orbitals in the oxygen atoms with the *d*‐orbitals of the multivalent metals introduce supplementary opportunities for fine‐tuning the electronic structure within the high‐entropy system (Figure S19, Supporting Information). As can be seen from the total density of states (DOS) in **Figure** **3**a, the bandgap of La_2_(CNMZNL)RuO_6_ is shortened to a level of 2.11 eV compared to that of La_2_(CNMZ)RuO_6_ (2.23 eV), explaining why the introduction of the AM^+^ enhances the conductivity and electron flow. The appearance of a characteristic impurity energy level at the bandgap may also reflect the strong electron mobility. By further comparing their *d*‐band centers in key metal elements, we observed from Figure 3b that the DOS of the Ru 4*d* orbitals in La_2_(CNMZNL)RuO_6_ is farther away from the Fermi level (−1.91 eV) compared, to the La_2_(CNMZ)RuO_6_ system (−1.67 eV), suggesting the loss of its charge. Instead, the d‐band centers of the Co 3*d* and Ni 3*d* orbitals shift toward the Fermi level, implying their potential to act as charge reservoirs (Figure 3c,d). Bader charge analysis was performed to verify these electron transfer processes, as shown in Figure 3e–g. The calculations show that the Ru, Co, and Ni atoms in La_2_(CNMZNL)RuO_6_ carry a charge of +2.08 |e|, +1.37 |e|, and +1.27 |e|, respectively, while in La_2_(CNMZ)RuO_6_ the charges are +2.00 |e|, +1.41 |e| and +1.29 |e|, respectively. This indicates that the electron transfers from Ru to Co and Ni in La_2_(CNMZNL)RuO_6_, are in agreement with the XAS results and DOS analysis. Moreover, Bader charge analysis reveals a decrease in the surface valence state of metals compared to their bulk valance state, due to the disruption of the atomic bonding at the surface, resulting in coordination unsaturation (details in Figure S20, Supporting Information). Essentially, the AM^+^‐induced super‐exchange interaction enriches the complexity of the energy bands and orbital hybridization, thus paving the way for promising catalytic platforms endowed with a diverse array of electronic structures.

Guided by cutting‐edge theoretical analysis, the UV–vis absorption spectra of La_2_(CNMZNL)RuO_6_ and related materials reveal critical insights into their electronic behavior, with optical bandgaps calculated using the Tauc equation. The broad absorption band ≈378 nm indicates a *p*–*d* charge transfer transition [O(2*p*) → high‐entropy sites(3*d*)/Ru(4*d*)] within the octahedral centers of La_2_(CNMZNL)RuO_6_ (Figure S21a, Supporting Information).^[^ ^59^ ^]^ This peak shift phenomenon is attributed to band bending facilitated by enhanced charge transfer via lattice oxygen and octahedral distortion.^[^ ^60^ ^]^ Upon the introduction of AM^+^ into its lattice matrix, the bandgap of La_2_(CNMZNL)RuO_6_ contracts from 4.23 to 4.02 eV, enhancing charge carrier excitation to the conduction band and thus improving the electrical conductivity of La_2_(CNMZ)RuO_6_ in Figure 3h.^[^ ^61^ ^]^ Further insights into the electron band configuration were provided by valence band XPS (VB‐XPS) analysis. Figure 3i displays the VB‐XPS spectra, elucidating an energy edge at 1.35 eV for La_2_(CNMZNL)RuO_6_, while the VB maximum of La_2_(CNMZ)RuO_6_ shifts to 0.17 eV. The absence of oxygen‐deficient structures was confirmed via electron paramagnetic resonance (EPR) (Figure S21b, Supporting Information), indicating that the incorporation of AM^+^ did not disrupt the conventional crystal lattice or introduce oxygen vacancies, consistent with previous structural assessments and O 1*s* XPS electronic characterization. Charge redistribution facilitated by the super‐exchange interaction thus directly results in a noticeably reduced bandgap at the Fermi level, as illustrated in Figure 3j. This phenomenon likely stems from the complex orbital hybridization of polymetallic elements and the subsequent modifications in the coordination environment.

### 2.4 Comprehensive Evaluation of HER Performance

**Figure 4:**
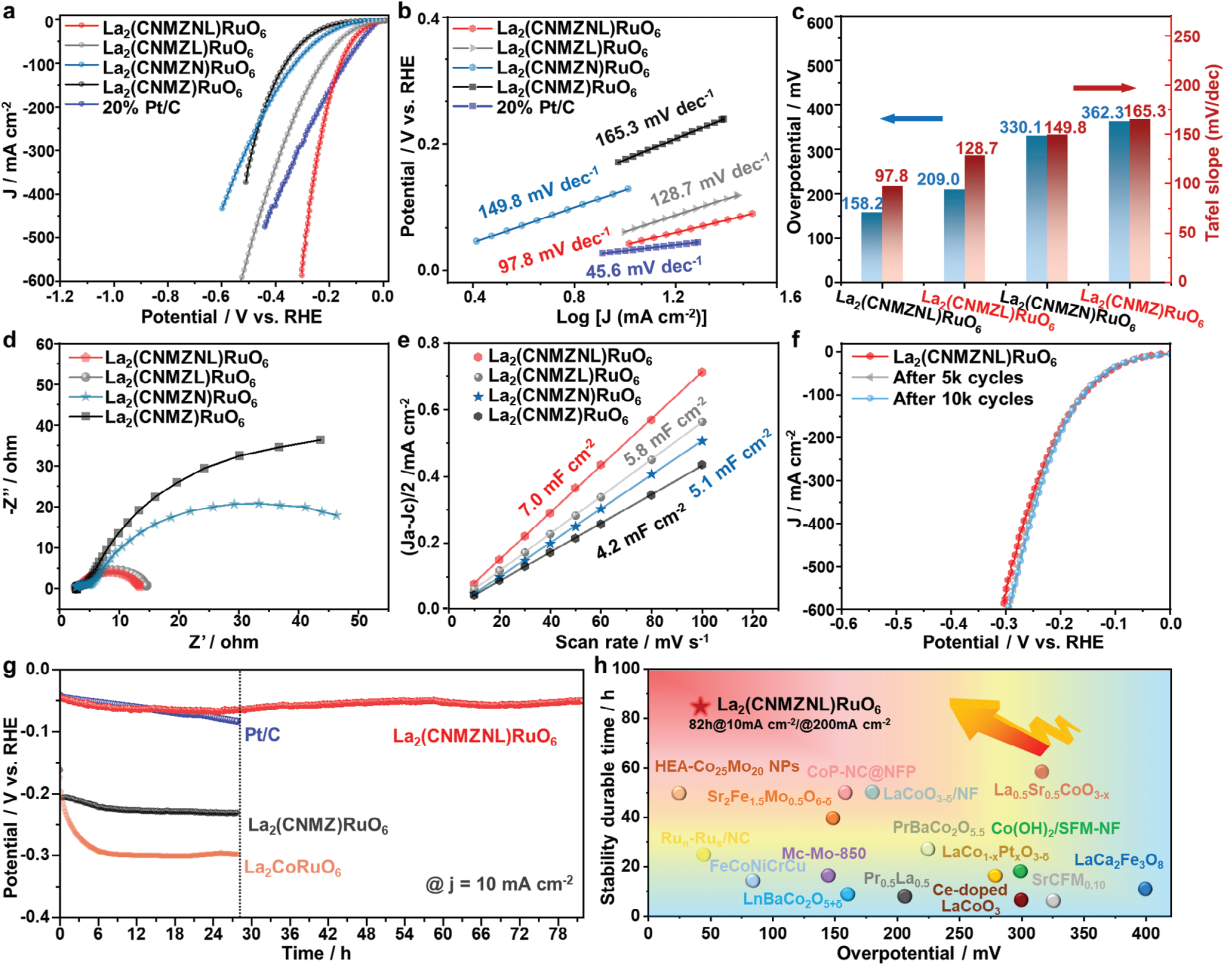
The electrochemical performance of four high entropy systems and Pt/C. a) Linear sweep voltammetry (LSV) curves of the electrocatalytic activity. b) Tafel slopes. c) The overpotential and Tafel slope of different concentrations (*j* = 100 mA cm^−2^). d) EIS. e) Evolution of *j* as a function of scan rate. f) ADT test. g) Stability measurement of the electrode under a constant current of 10 mA cm^−2^. h) Overpotentials (left) and the stability durable time (right) of La_2_(CNMZNL)RuO_6_ and other recently reported high‐performance HER catalysts. The perovskite, alloy compounds, and high entropy materials in similar systems are mainly listed.

The electrocatalytic HER activity of the La_2_(CNMZNL)RuO_6_ and other related samples was rigorously assessed using a three‐electrode setup in a 1 m KOH solution at room temperature. Initial cyclic voltammetry (CV) was employed to stabilize the performance, ensuring accurate electrochemical testing results. For a fair comparison, the catalytic performance of the samples is evaluated at identical current densities. Initially, the alkaline HER performance of the unmodified single‐phase Ru‐based double perovskite, without employing an entropy‐driven strategy, was evaluated in Figure S22 (Supporting Information), revealing inadequate electrochemical properties. Interestingly, upon increasing the configuration entropy to form the La_2_(CN)RuO_6_ sample, the synergistic effect of Co^2+^, Ni^2+^, and Ru^4+^ potentially optimized the active sites, leading to a notable decrease in overpotential. To augment the level of configuration entropy and achieve pure phase structures, Mg^2+^ and Zn^2+^ were introduced. This step aimed to enhance catalytic stability, although it could potentially lead to partial coverage of active sites and a subsequent reduction in conductivity. Subsequently, more electrochemical evaluations are focused on the polarization current curves of La_2_(CNMZNL)RuO_6_, La_2_(CNMZN)RuO_6_, La_2_(CNMZL)RuO_6_, and La_2_(CNMZ)RuO_6_ samples. These results obviously demonstrate that the incorporation of AM^+^ can significantly improve catalytic performance. As depicted in **Figure** **4**a, the polarization curves of the samples were corrected for both current and resistance (IR) at a level of 90%. The reference electrode of Hg/HgO was pre‐calibrated before the measurement. It is evident that the La_2_(CNMZNL)RuO_6_ exhibits a reduced overpotential (40.7 mV) at a current density of 10 mA cm^−2^. Additionally, La_2_(CNMZNL)RuO_6_ demonstrates a Tafel slope of 97.8 mV dec^−1^ (Figure 4b), indicating efficient HER kinetics on its catalysis. Both the overpotential and Tafel slope values for La_2_(CNMZNL)RuO_6_ are lower than those of other samples (Figure 4c), highlighting their superior catalytic properties. Moreover, when compared with other advanced pure‐phase perovskite catalysts reported in recent literature (Table S6, Supporting Information), La_2_(CNMZNL)RuO_6_ stands out as one of the best electrocatalysts for HER, with an exceptionally low overpotential. To gain deeper insights into the electrode kinetics during HER catalysis, electrochemical impedance spectroscopy (EIS) was utilized, as shown in Figure 4d. The Nyquist plots displayed two semicircles, and an equivalent circuit model was employed to fit the data, extracting the charge transfer resistance (R_ct_). The R_ct_ for La_2_(CNMZNL)RuO_6_ is determined to be 0.96 Ω, which is superior to other samples, as listed in Table S7 (Supporting Information). This lower charge transfer resistance indicates that La_2_(CNMZNL)RuO_6_ facilitates faster and more efficient charge transfer during HER electrocatalysis. The electrochemical surface areas (ECSA) of all samples were estimated from the double layer capacitance (C_dl_), inferred from cyclic voltammetry (CV) curves recorded at various scan rates (Figure S23, Supporting Information). The plot of (*J*_a_ − *J*_c_)/2 versus scan rate for all samples is shown in Figure 4e, with ECSA values calculated from the slopes of C_dl_. La_2_(CNMZNL)RuO_6_ exhibits the largest ECSA, which implies that the introduction of Li^+^ and Na^+^ creates a significant number of active sites. This increase in active sites can be attributed to the partial lattice distortion induced by the high‐entropy system and the changes in the coordination environment caused by AM^+^. The suspension of certain coordination bonds may activate both the active sites and the active regions, consistent with the alterations in the coordination environment observed in the XAS spectra.

On the contrary, both the elevated and decreased AM^+^ content in La_2_(CNMZNL)RuO_6_ resulted in decreased catalytic performance, indicating that suitable AM^+^ content can serve as a reliable quantitative indicator for performance. Excessive addition may lead to diminished conductivity or compromised proton transfer capability (Figure S24, Supporting Information). Furthermore, insignificant super‐exchange effects arising from an excessive decrease in AM^+^ can result in the reduction of high catalytic activity at the active sites. Meanwhile, the incorporation of AM^+^ into the single‐component double perovskite showed an indeterminate effect on electrocatalytic performance, highlighting the complex cocktail effect within the high‐entropy system that enhances the activation of AM^+^ for its utility (Figures S25–S27, Supporting Information). Overall, these results suggest that the AM^+^‐induced super‐exchange interaction significantly enhances charge transfer and dynamic activation. This enhancement promotes the charge transport capacity and increases the number of active sites for HER, leading to improved electrocatalytic performance.

To evaluate the practicality of the catalyst, stability represents another crucial parameter. As shown in Figure 4f, during the accelerated degradation test (ADT) spanning 5000 and 10 000 cycles between 0 and −0.25 V (vs RHE) at 100 mV s^−1^, the polarization curve of the La_2_(CNMZNL)RuO_6_ catalyst demonstrates no discernible negative shift. Furthermore, the La_2_(CNMZNL)RuO_6_ catalyst exhibits stability for up to 82 h at room temperature in 1 m KOH solution at 10 mA cm^−2^ (Figure 4g). In contrast, the commercial Pt/C, the medium entropy material La_2_(CNMZ)RuO_6_, and the unit perovskite La_2_CoRuO_6_ experience degradation of 106.5%, 45.8%, and 69.7%, respectively, over 27 h of voltage–time (VT) testing relative to their initial potentials. Notably, the 82‐h VT test also shows virtually negligible attenuation at a current density of 200 mA cm^−2^, demonstrating its potential for industrial utilization (Figure S28, Supporting Information). Excitingly, it must be mentioned that the La_2_(CNMZNL)RuO_6_ catalyst significantly demonstrates its fantastic HER catalytic activities and robust stability, in comparison to the reported electrocatalysts including typical perovskite, high‐entropy materials and alloys in Figure 4h. It confirms the efficacy of the approach involving the incorporation of AM^+^ and the establishment of an entropy‐driven platform. The stability of La_2_(CNMZNL)RuO_6_ was further substantiated by the post‐characterization of the utilized catalyst. After the durable HER stability test, the XRD pattern reveals that the La_2_(CNMZNL)RuO_6_ retained a pristine structure, akin to its pre‐reaction state (Figure S29a, Supporting Information). Raman spectroscopy shows that the target sample exhibits virtually no phase transition or strain at the molecular scale, as the peak position and width of the Raman active mode remain virtually unchanged (Figure S29b, Supporting Information). SEM and TEM were conducted to evaluate the morphology subsequently after enduring HER stability test. The morphology of La_2_(CNMZNL)RuO_6_ implies that the morphology remains unchanged at the nanoparticle size (Figures S30 and S31a, Supporting Information). The SAED and HRTEM reveal a single‐phase crystalline perovskite structure of the sample post‐VT, thereby indicating the structural stability of the La_2_(CNMZNL)RuO_6_ (Figure S31b,c, Supporting Information). STEM‐mapping and EDS‐mapping in Figure S31d,e (Supporting Information) show the uniform distribution of all elements. However, following the VT reaction of La_2_(CNMZ)RuO_6_, HR‐TEM reveals the formation of Ru clusters on its surface, and STEM mapping also illustrates the segregation of Ru elements and the formation of Ru clusters in Figure S32 (Supporting Information). The XPS spectra were obtained, revealing similar deconvoluted peaks as observed in the spectra before testing (Figure S33, Supporting Information). For instance, under prolonged reduction potentials, both the relative peak area of Co^0^ species and the surface chemical states of Ru remain nearly constant. This stability is likely due to the structural integrity of the high‐entropy solid solution, with other metals providing protection to the active elements by undergoing reduction themselves. Moreover, ICP‐MS analysis indicates that the limited dissolution observed at different time points and current densities confirms the catalyst's ability to sustain consistent performance over time (Table S8, Supporting Information). Therefore, the reason for the maintenance of the properties of high‐entropy perovskite materials is due to the maintenance of an unchanged crystalline phase and the absence of elemental segregation under the high‐entropy effect facilitated by AM^+^. It is important to note that the cocktail effect of the increase in configuration entropy and the introduction of AM^+^ results (Figure S27, Supporting Information) in a high degree of stability during the test.^[^ ^62^, ^63^ ^]^ Equally significant is the unit‐cell stability of the RuO_6_ and HEO_6_ octahedra, alongside the constancy of the molecular level and the similar electronic structure, which furnish a stable platform for efficient catalytic hydrogen evolution. Moreover, this underscores a pivotal notion for ensuring the structural stability and crystal regulation of pure phase materials.

### 2.5 DFT Calculations and Electrocatalysis Mechanism

**Figure 5:**
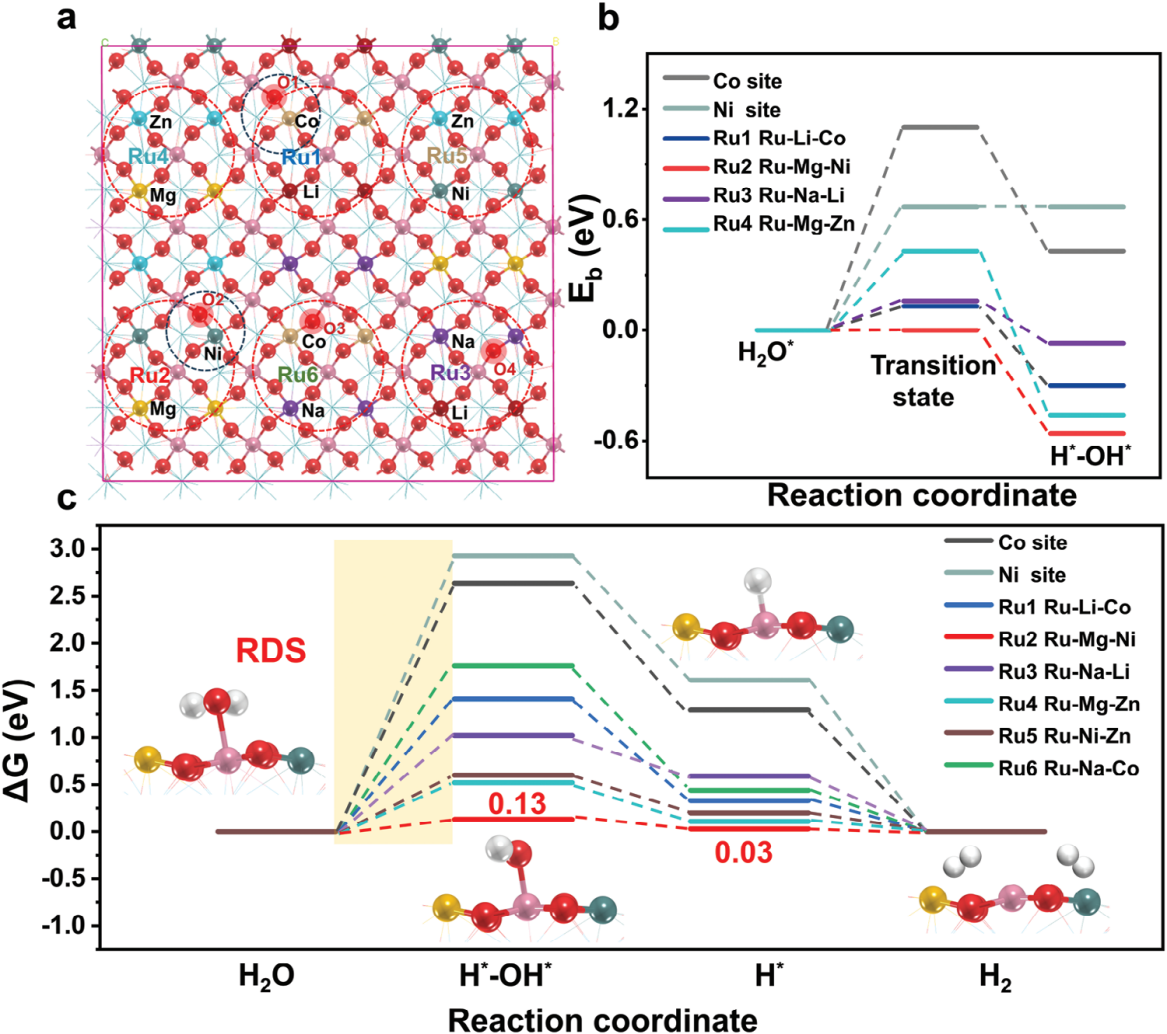
a) Computational models and selected computational sites. b) Energy profiles for the water‐dissociation process on Co, Ni, and four Ru sites. c) Gibbs free energy diagrams of alkaline HER where the dissociated H is adsorbed on a metal site. The yellow highlight is the decisive speed step. The models in the figure show the schematic of the synergistic alkaline HER catalysis mechanism on La_2_(CNMZNL)RuO_6_.

The remarkable improvement in the alkaline HER activity on the HEDP catalyst was elucidated by systematic DFT calculations using the La_2_(CNMZNL)RuO_6_ (001) as a surface model. Note that the optimal structural configuration of La_2_(CNMZNL)RuO_6_ is determined by the lowest total energy among all possible structures. Twelve potential active sites involving Ru/Co/Ni/O elements were considered to calculate the alkaline HER process, including H_2_O dissociation, OH desorption, and H_2_ production (**Figure** **5**; Figure S34, Supporting Information). The calculations indicate that Ru sites exhibit the lower energy barrier of H_2_O dissociation (≤0.43 eV) compared to Co and Ni sites (1.10 eV, 0.67 eV); especially on the Ru2 site that are adjacent coordinated with Mg and Ni, H_2_O dissociation becomes a barrierless process (Figure 5b). From a kinetic perspective, it can be concluded that the Ru sites may serve as the potential active site for H_2_O dissociation.

Furthermore, the Gibbs free energy profiles for the alkaline HER process on twelve active sites are illustrated in Figure 5c and Figure S34 (Supporting Information), with the aim of comprehensively elucidating its limiting step and the optimal active site. It can be seen that the potential‐limiting step on eight active sites is the dissociation of H_2_O where the dissociated H is adsorbed on a metal site (referred to as the Volmer step) due to its endothermicity (Figure 5c). Notably, the Ru2 site exhibits the lowest overpotential of 0.13 eV, confirming its role as the active site. This is consistent with the DOS result, which indicates that the *d*‐band center of Ru is closer to the Fermi level (Figure 3e–g), thereby improving the adsorption of intermediates. We also compared the condition where the dissociated H is adsorbed on the lattice oxygen and found that they have an overpotential greater than 1 eV due to the limitation of H desorption (Figure S34, Supporting Information). In summary, the mechanism of the alkaline HER on La_2_(CNMZNL)RuO_6_ perovskite oxide has been elucidated. Essentially, the Ru site, which exhibits minimal kinetic barriers, displays favorable attributes for both OH^−^ desorption and H_2_ production, rendering it an efficient active site for promoting HER under alkaline conditions. This tendency may stem from charge flow facilitated by super‐exchange interaction and the intricate orbital hybridization coupling mechanism among the multi‐metals of high‐entropy materials. Within this intricate charge‐coupling milieu, the reduction of the bandgap and the enhancement of mass transfer dynamics synergistically facilitate efficient reactions.

## 3 Conclusion

In summary, a series of pure‐phase HEDP catalysts are proposed to explore their catalytic activity for hydrogen evolution by introducing the AM^+^ elements and employing nano‐level design in an alkaline solution. The synthesis strategy involving a solid‐state reaction and ball‐milling leads to the formation of uniform HEDP nanocrystals. The La_2_(CNMZNL)RuO_6_ electrocatalyst demonstrates excellent HER activity, achieving overpotentials of 40.7 and 158.2 mV versus RHE for current densities of *j* = −10 mA cm^−2^ and *j* = −100 mA cm^−2^, respectively. Moreover, La_2_(CNMZNL)RuO_6_ exhibits outstanding stability with no degradation after 10 000 cycles and maintained superior performance for over 82 h at a current density of 10 mA cm^−2^, surpassing the catalytic performance of most currently reported pure‐phase perovskite materials. We elucidate that the La_2_(CNMZNL)RuO_6_ plays a pivotal role in enhancing the HER catalytic activity due to several factors: i) Introducing AM^+^ elements into the solid solution modifies the local coordination environment of the HEDP family, facilitating tunability of multiple overlapping orbitals. Moreover, a high‐entropy strategy ensures prolonged service life in alkaline environments. ii) The super‐exchange interaction enhances the orbital hybridization of polymetallic elements within high‐entropy systems, narrowing the bandgap and improving both electrical conductivity and mass transfer capability within the system. iii) Electron reflux via super‐exchange interaction further promotes the electron transitions among Ru, Co, Ni, and O elements, modifying the capability of the Ru active site to dissociate water intermediates, thus facilitating the dissociation of OH^*^ and the production of H_2_. Just as the tip of the iceberg, this new mechanism driven by the super‐exchange interaction, provides a novel pathway for researching renewable energy applications of high‐entropy perovskite materials. It also offers fresh impetus for further exploration of the super‐exchange interaction mechanism, promising advancements in the field of catalysis and renewable energy.

## 4 Experimental Section

### Materials

Lanthanum oxide (La_2_O_3_, 99.9% Aladdin), ruthenium oxide (RuO_2_, 99.9%, Aladdin), cobalt(III) oxide (Co_2_O_3_, 99.99%, Macklin), nickel(III) oxide (Ni_2_O_3_, 99%, Innochem), magnesium oxide (MgO, 99.99%, Innochem), zinc oxide (ZnO, 99.99%, Innochem), sodium carbonate (Na_2_CO_3_, 99.999%, Aladdin), lithium carbonate (Li_2_CO_3_, 99.999%, Aladdin), cupric oxide (CuO, 99.995%, Alfa), iron(III) oxide (Fe_2_O_3_, 99.9%, Innochem), and manganese oxide (Mn_2_O_3_, 98%, Innochem) were used as received without any further purification in this work.

### Synthesis of Materials

All samples with different structural entropies were prepared by a modified solid‐state and ball‐milling method. First, the high‐purity individual elements were weighed according to the nominal ratio, and then ground to be mixed uniformly. After pressing them into sheets, they are sintered in a muffle furnace using 900–1000 °C. It should be noted that materials containing Na and Li should be sintered into phases at 900 degrees and sealed to prevent the escape of alkali metal elements. Subsequently, 500 mg of the sample obtained from sintering was removed and ball milled in a high‐energy ball mill for 3 h to obtain high entropy nanocrystals.

### Material Characterizations

Morphology of the samples was observed on field‐scanning transmission electron microscopy (TEM) images acquired on a JEM‐ARM200F transmission electron microscope operated at 200 kV and scanning transmission electron microscopy (FE‐SEM, S‐4800, Japan) equipped with energy dispersion spectra (EDS) and elemental mapping. Powder XRD patterns were acquired at room temperature, using an X‐ray diffractometer (D/max 2500 V) attached with Cu Kα radiation at an operating voltage and current of ≈40 kV and 150 mA, respectively. Rietveld refinements for XRD were conducted to obtain detailed structural and compositional information using GSAS (II) software. The element contents in the catalysts were determined by an Inductively Coupled Plasma Optical Emission Spectrometer (ICP‐OES) on an Agilent 5110. XPS measurements were obtained using an X‐ray photoelectron spectrometer (Escalab 250Xi) equipped with an Al Kα radiation source (1487.6 eV) and a hemispherical analyzer with a pass energy of 30.0 eV and an energy step size of 0.05 eV. The binding energy of the C 1*s* peak at 284.8 eV was considered as an internal reference. Spectral deconvolution was performed by Shirley background subtraction by using a Voigt function convoluting the Gaussian and Lorentzian functions. Ru‐*K* edge, Co‐*K* edge, Ni‐*K* edge, and Zn‐*K* edge, hard XAS spectra were collected at the TPS‐44A beamline of the National Synchrotron Radiation Research Center (NSRRC, Hsinchu, Taiwan).

### Electrocatalytic Measurements

For the preparation of catalyst ink, all catalyst powders were dispersed into a mixture solution (200 µL of ethanol, 800 µL of ultrapure water), respectively. The catalyst was ultrasonically dispersed in ink. The obtained catalyst inks were uniformly loaded onto freshly carbon paper electrodes (0.5 × 0.5 mm) to form the work electrode. The amount of deposited catalyst was calculated to be ≈250 µg and the loading on the working electrode was controlled to be 1 mg cm^−2^. The HER performance of these samples was characterized by a CHI 760 potentiostat with a three‐electrode system in 1.0 m KOH. All polarization curves were collected at a scan rate of 5 mV s^−1^ and calibrated to the reversible hydrogen electrode according to the following equation: *E*_RHE_ = *E*_Hg/HgO_ + 0.098 + 0.059 ^*^ pH. An IR compensation level of 90% based on the electrochemical impedance spectroscopy. ECSA analysis was used CVs taken at various scan rates (10–100 mV s^−1^) were collected and used to extract the double‐layer capacitance. The EIS measurements for HER were performed at a 100 mV overpotential under the influence of an alternating current (ac) voltage of 50 mV from 100 to 0.1 Hz. In addition, accelerated durability tests (ADT) were carried out at the voltage range of 0 to −0.25 V (vs RHE) for 10 000 cyclic voltammetry cycles with a scan rate of 100 mV s^−1^ in 1.0 m KOH. Chronopotentiometric tests at the continuous current density of 10 and 200 mA cm^−2^ for HER were conducted to explore the stability of the electrocatalysts.

### Computational Methods

The spin‐polarized density functional theory (DFT) calculations were performed by VASP,^[^ ^64^ ^]^ using the Perdew–Burke–Ernzerh (PBE) functional within the generalized gradient approximation (GGA).^[^ ^65^ ^]^ The core–valence electron interaction was described by the projector augmented wave (PAW) method,^[^ ^66^ ^]^ and the valence electronic states were expanded in the plane–wave basis sets with a cutoff energy of 400 eV. Based on the experimental characterization that the doped metals were in equal proportions in the B' position of perovskite oxide, the La_2_(CNMZNL)RuO_6_ and La_2_(CNMZ)RuO_6_ were modeled by integrating three and two LaRuO_3_ bulks, respectively, as a structural template, and the corresponding optimal configurations were determined by comparing the total energies of 120 and 6 bulk structures generated by random combination among metal elements. La_2_(CNMZNL)RuO_6_(001) with six atomic layers were applied to calculate the catalytic mechanism of alkaline HER, and the vacuum between the slabs was 15 Å to exclude the mutual influence. For these surface slabs, a 1 × 2 × 1 K‐point mesh was used, respectively. All geometric structures were relaxed until the energy and forces converged to 10^−5^ eV and 0.05 eV Å^−1^, respectively. van der Waals dispersion (DFT‐D3) was included, and the DFT + *U* approach with the on‐site coulomb correction included was applied to describe the localized *d* and *f* orbitals of Co Ni, Ru, La elements (3*d* for Co: *U*_eff_ = 3.30 eV, 3*d* for Ni: *U*_eff_ = 6.40 eV, 4*d* for Ru: *U*_eff_ = 2.02 eV, 4*f* for La: *U*_eff_ = 6.00 eV).^[^ ^67^, ^68^, ^69^ ^]^ The Gibbs free energy changes (Δ*G*) of each elementary reaction for alkaline HER were calculated using the computational hydrogen electrode model, which was proposed by Nørskov's group.^[^ ^70^ ^]^ The Δ*G* values on various surfaces were calculated with the equation: Δ*G* = Δ*E*_ads_ + Δ*E*_ZPE_ – TΔ*S*, where Δ*E*_ads_ is the adsorption energy of reaction intermediates; Δ*E*_ZPE_ and Δ*S* are the energy differences in zero‐point energy and entropy respectively.

## Conflict of Interest

The authors declare no conflict of interest.

## Supporting information

Supporting Information

## Data Availability

The data that support the findings of this study are available from the corresponding author upon reasonable request.
