# Supplementary material for: Alkali Metals Activated High Entropy Double Perovskites for Boosted Hydrogen Evolution Reaction
Source: Adv Sci (Weinh). 2024 Sep 9;11(42):2406453. doi: 10.1002/advs.202406453 (PMC11558161; doi:10.1002/advs.202406453)
Supplement: Supplementary file 1 — Supporting Information [file ADVS-11-2406453-s001.docx]

Supporting Information

**Alkali Metals Activated High Entropy Double Perovskites for Boosted Hydrogen Evolution Reaction**

Ning Sun^†^, Zhuangzhuang Lai^†^, Wenbo Ding, Wenbo Li, Tianyi Wang, Zhichuan Zheng, Bowen Zhang, Xiangjiang Dong, Peng Wei, Peng Du, Zhiwei Hu, Chih-Wen Pao, Wei-Hsiang Huang, Haifeng Wang*, Ming Lei, Kai Huang*, Runze Yu*

N. Sun, W. Ding, T. Wang, Z. Zheng, P. Du, M. Lei, K. Huang

State Key Laboratory of Information Photonics and Optical Communications, School of Science, Beijing University of Posts and Telecommunications, Beijing 100876, China
E-mail: huang-kai@bupt.edu.cn (K. Huang)

N. Sun, W. Li, B. Zhang, X. Dong, P. Wei, R. Yu

Center for High Pressure Science and Technology Advanced Research, Beijing, China

E-mail: Runze.yu@hpstar.ac.cn (R. Yu)

Z. Lai, H. Wang

State Key Laboratory for Green Chemistry Engineering and Industrial Catalysis, Centre for Computational Chemistry and Research Institute of Industrial Catalysis, School of Chemistry and Molecular Engineering, East China University of Science and Technology, Shanghai, 200237, P. R. China.

E-mail: hfwang@ecust.edu.cn (H. Wang)

Z. Hu

Max Planck Institute for Chemical Physics of Solids, Nothnitzer Strasse 40, Dresden 01187, Germany

C.-W. Pao, W.-H. Huang

National Synchrotron Radiation Research Center, 101 Hsin-Ann Road, Hsinchu 300092, Taiwan, R.O.C

^†^These authors contributed equally.

This file includes:

Supplementary Figure 1-34

Supplementary Table 1-8

**Figures and Tables**

**
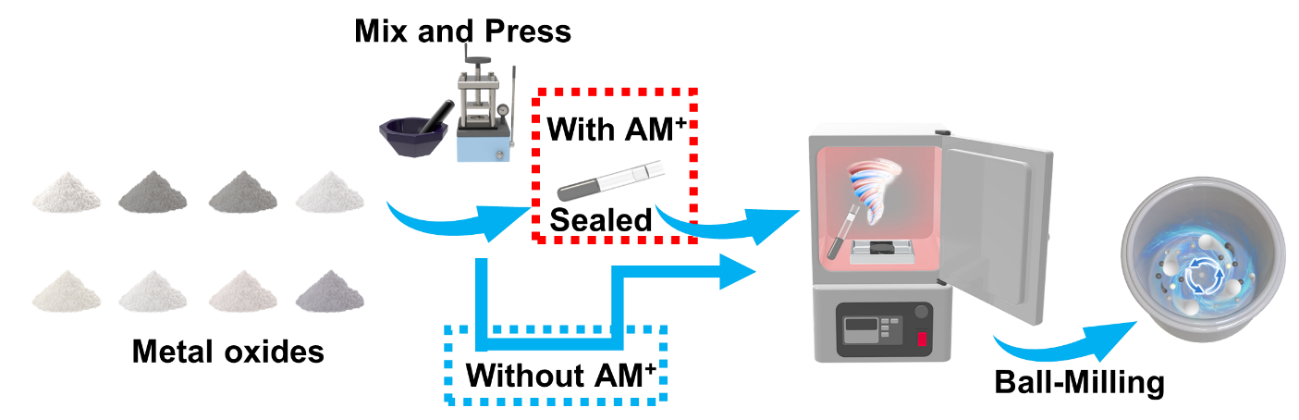
**

**Figure S1.** Schematic illustration for the synthetic process. The family of HEDPs composite was prepared via solid-state and ball milling treatment. In the introduction of AM^+^, it is necessary to pay attention to seal it into the quartz tube sintering, to avoid the escape of AM^+^.


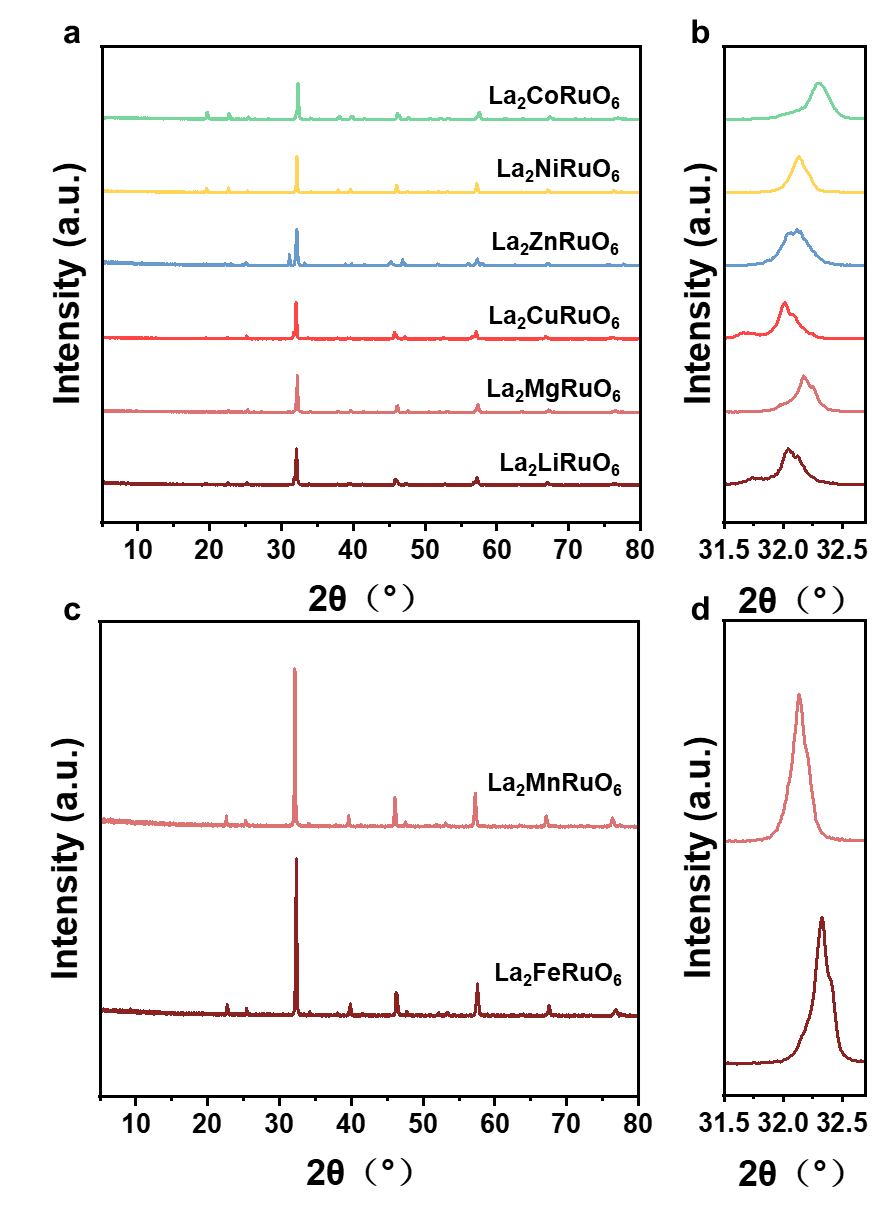


**Figure S2.** XRD pattern of unit double perovskite.

**
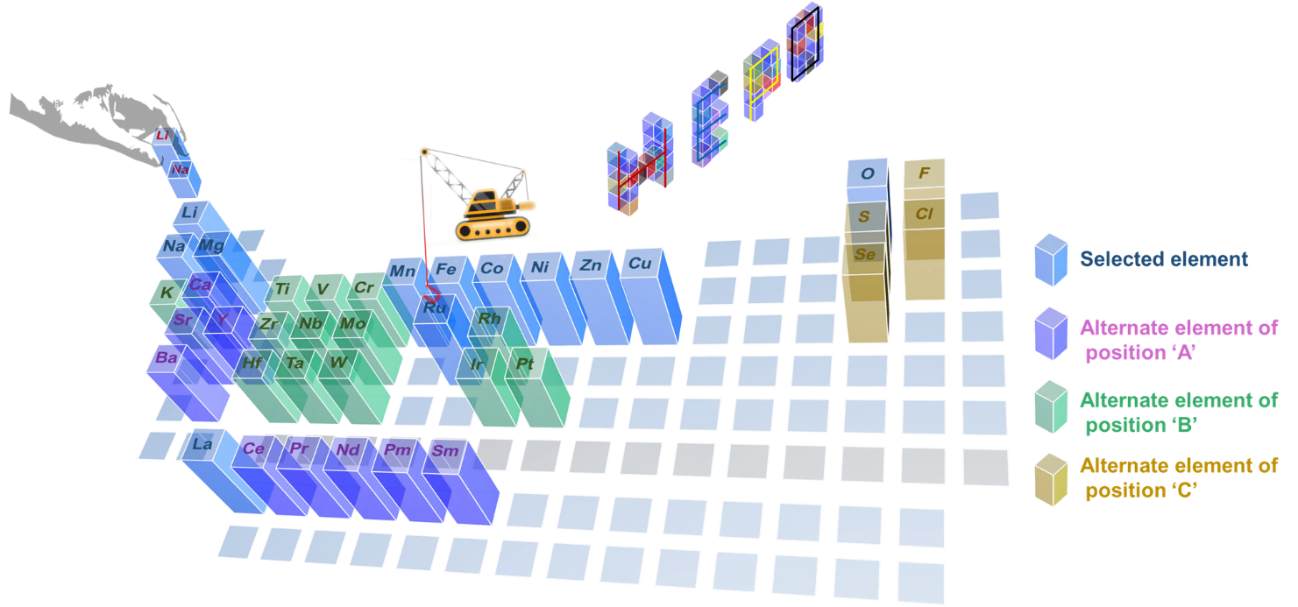
**

**Figure S3.** Schematic diagram about the elements selected by high-entropy double perovskite materials.


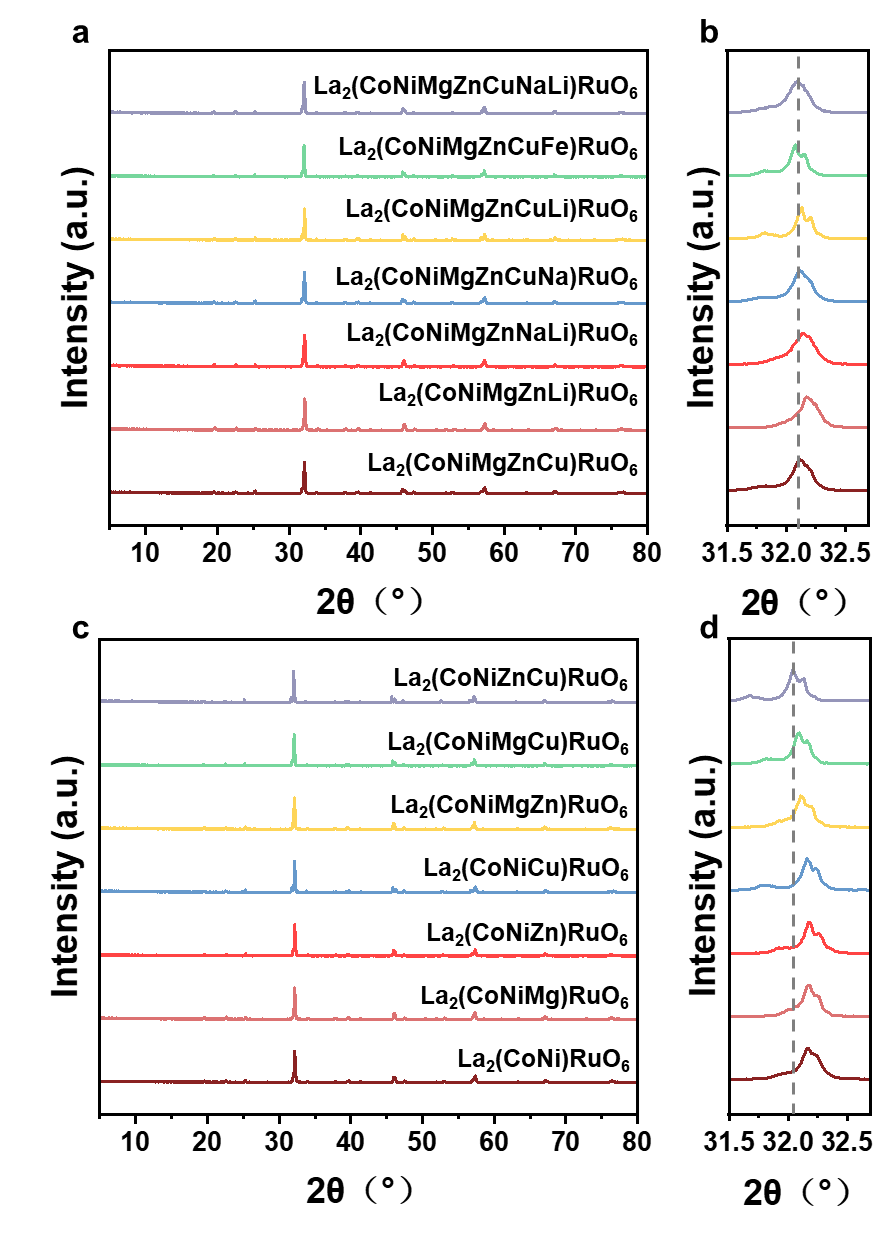


**Figure S4.** High entropy double perovskite and low configuration entropy double perovskite XRD patterns.


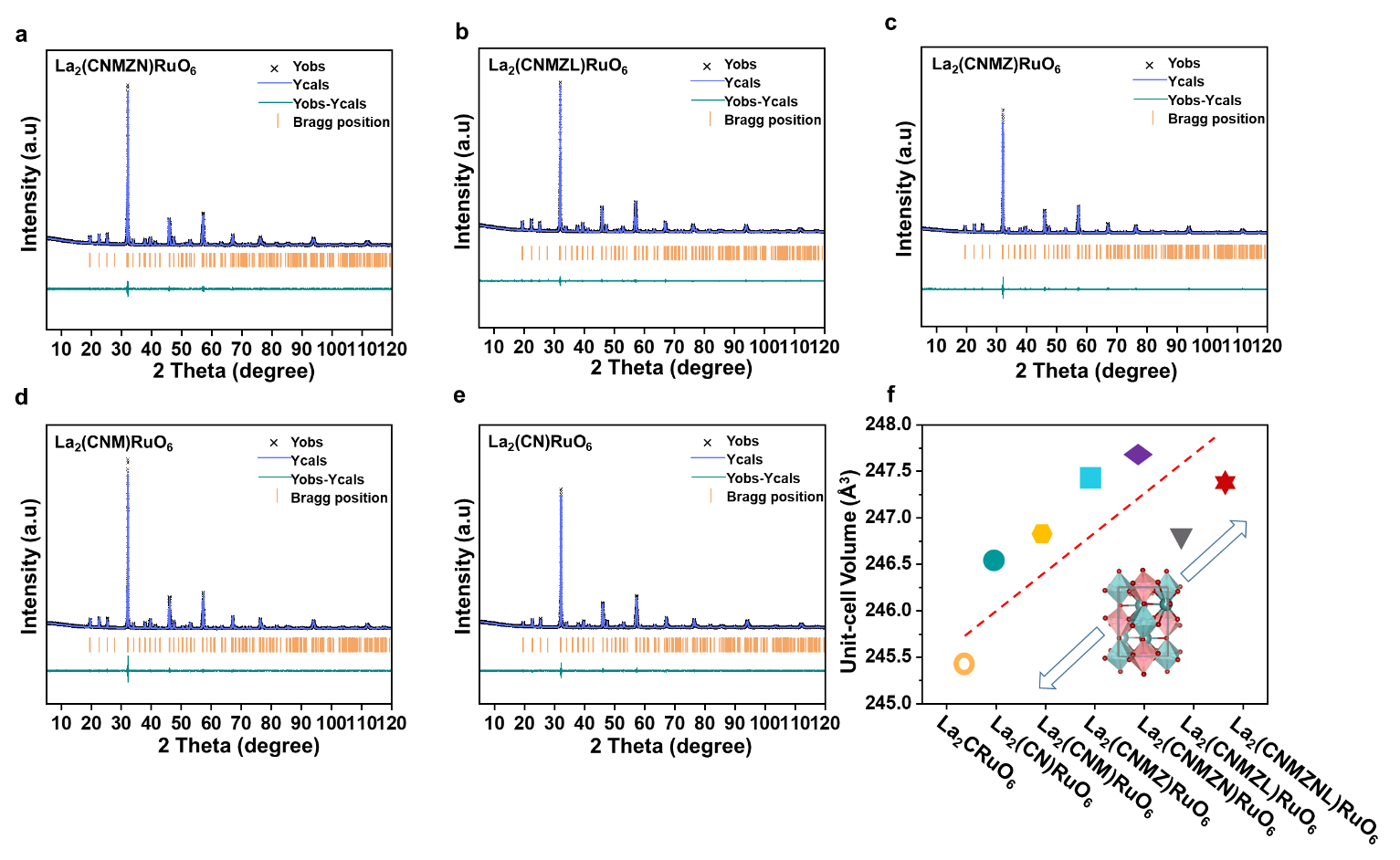


**Figure S5.** a)-e) XRD refinement of La_2_(CNMZN)RuO_6_, La_2_(CNMZL)RuO_6_, La_2_(CNMZ)RuO_6_, La_2_(CNM)RuO_6_ and La_2_(CN)RuO_6_. f) The relationship between the increase of configuration entropy and lattice size.


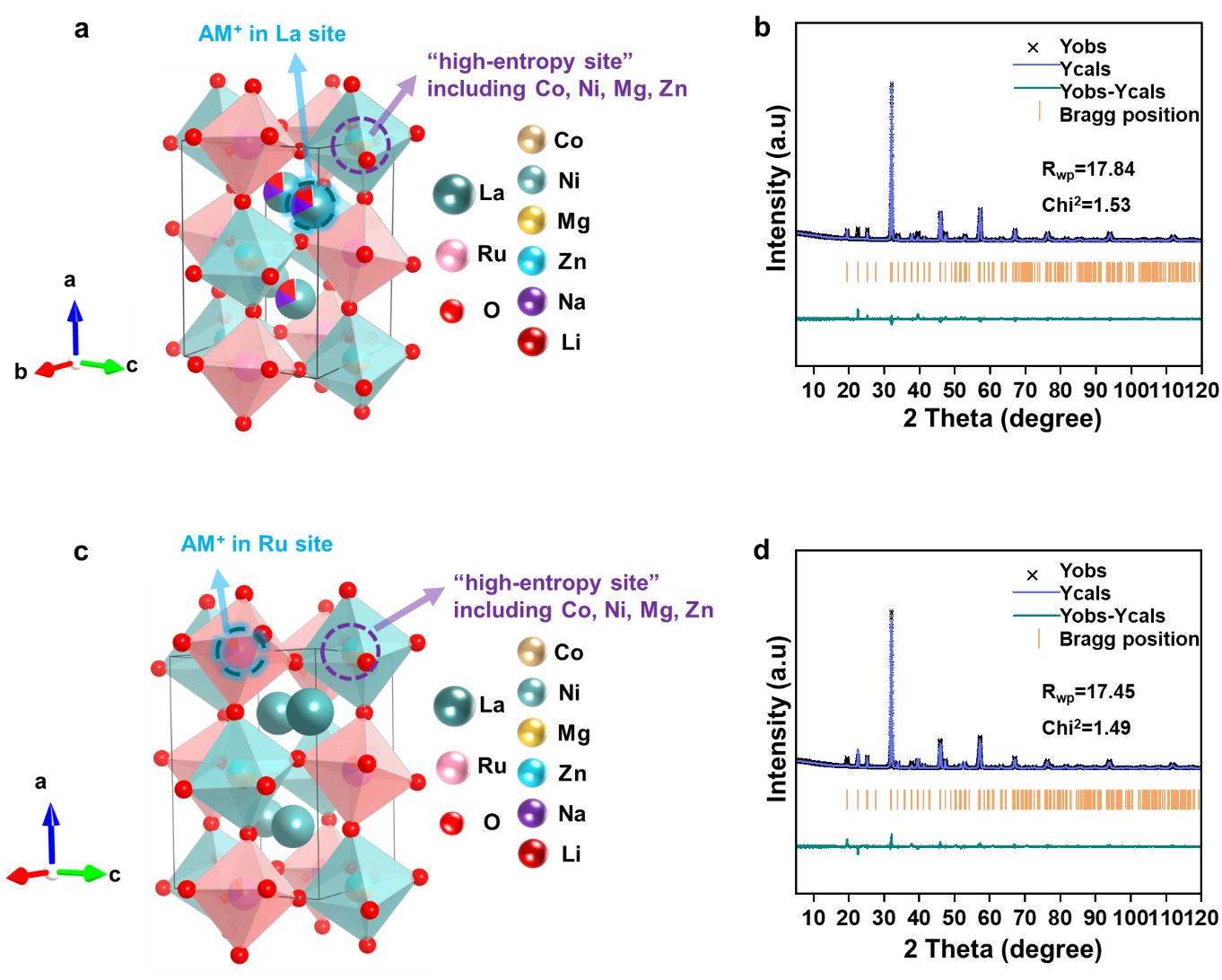


**Figure S6.** Schematic diagram of the model when AM^+^ and La element cooccupy the A site in A_2_B'B''O_6_ double perovkite crystal for a), and the corresponding XRD refinement for b). Schematic diagram of the model when AM^+^ and Ru element cooccupy the B'' site in A_2_B'B''O_6_ double perovkite crystal for c), and the corresponding XRD refinement for d). The refinement parameters of R_wp_ and Chi^2^ show a lower probability of AM^+^ sharing the A site with La or AM^+^ sharing the B' site with Ru compared to the high probability of AM^+^ sharing the B' site with other high entropy elements.

**
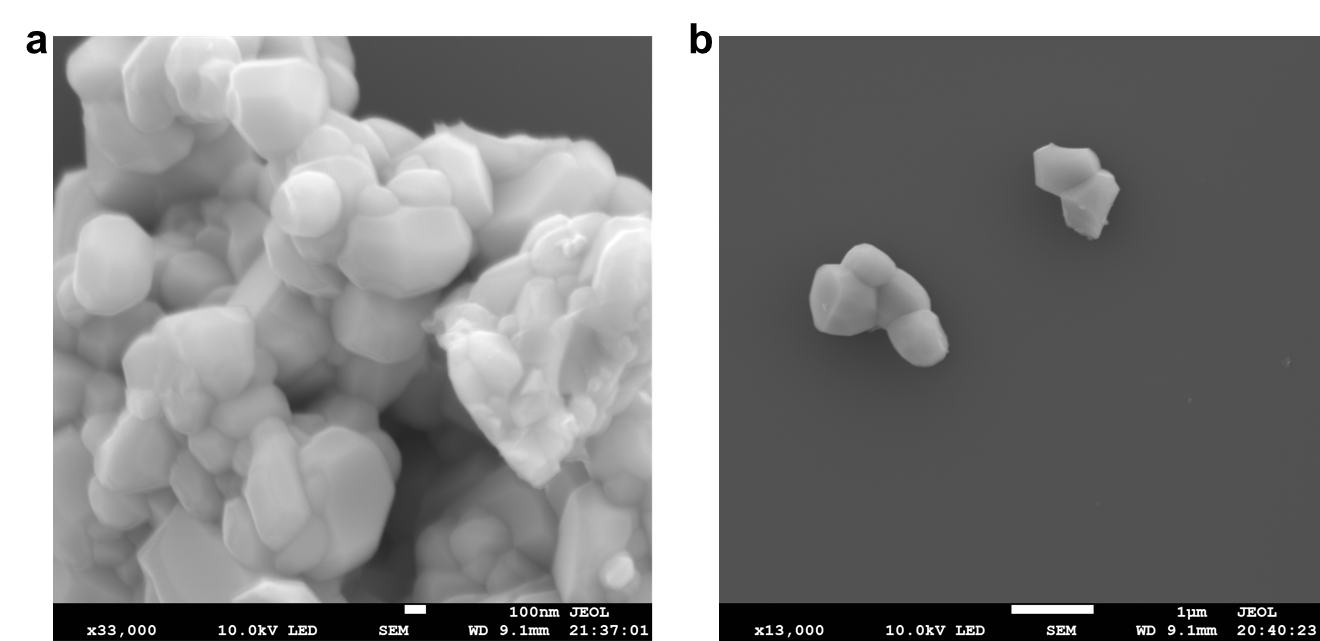
**

**Figure S7.** SEM of La_2_(CNMZNL)RuO_6_ before ball-milling.


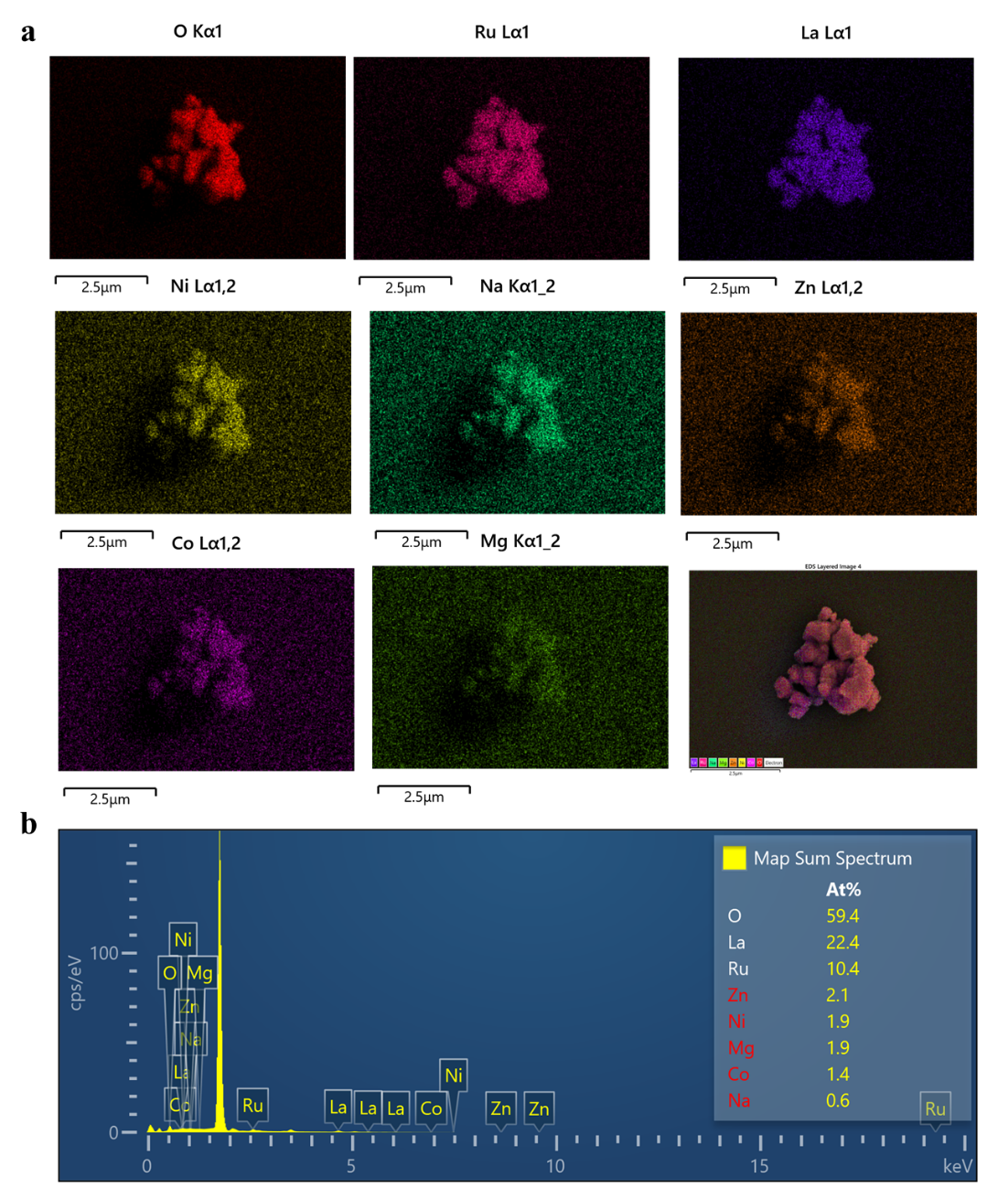


**Figure S8.** The SEM EDS-mapping of La_2_(CNMZNL)RuO_6_ and the corresponding element ratio.

**
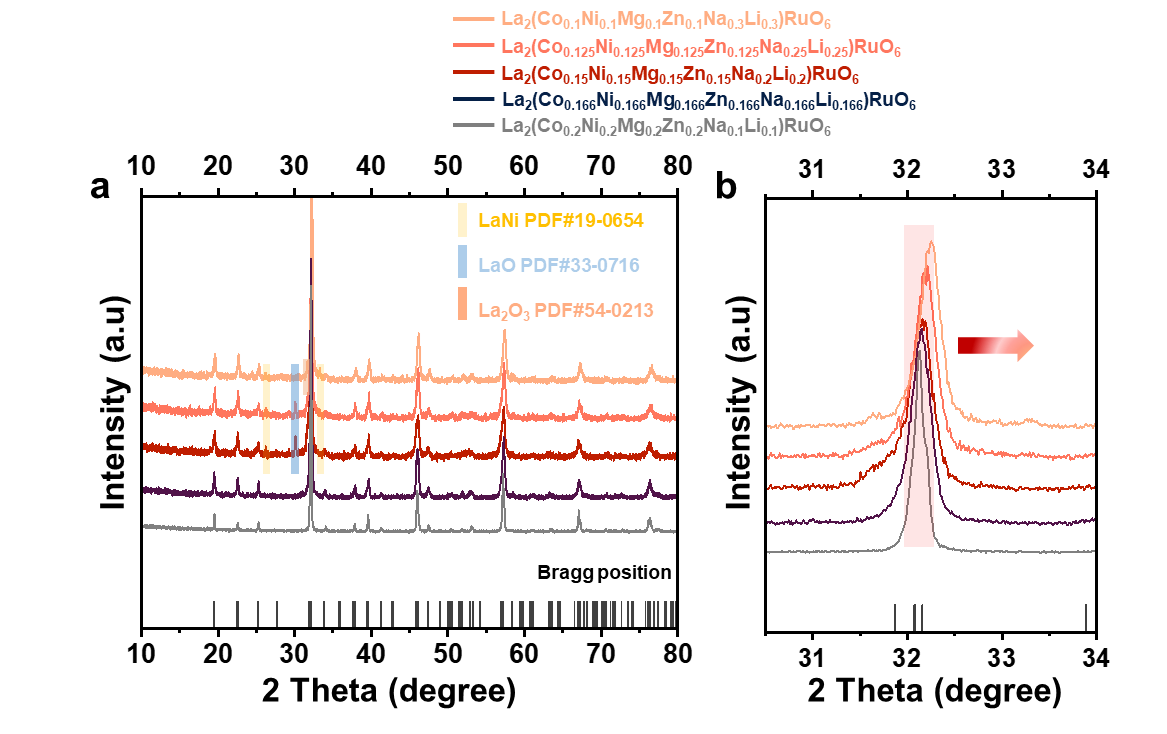
**

**Figure S9.** The XRD image with different content of AM^+^ for La_2_(Co_0.2_Ni_0.2_Mg_0.2_Zn_0.2_Na_0.1_Li_0.1_)RuO_6_, La_2_(Co_0.166_Ni_0.166_Mg_0.166_Zn_0.166_Na_0.166_Li_0.166_)RuO_6_, La_2_(Co_0.15_Ni_0.15_Mg_0.15_Zn_0.15_Na_0.2_Li_0.2_)RuO_6_, La_2_(Co_0.125_Ni_0.125_Mg_0.125_Zn_0.125_Na_0.25_Li_0.25_)RuO_6_ and La_2_(Co_0.1_Ni_0.1_Mg_0.1_Zn_0.1_Na_0.3_Li_0.3_)RuO_6_. Some of the heterogeneous phases are produced when nominal ratio of Na^+^ and Li^+^＞0.16. However, the main peak at about 32.2° still tends to shift to the right as the AM^+^ content increases, proving that more AM^+^ occupies the lattice sites to promote lattice contraction.


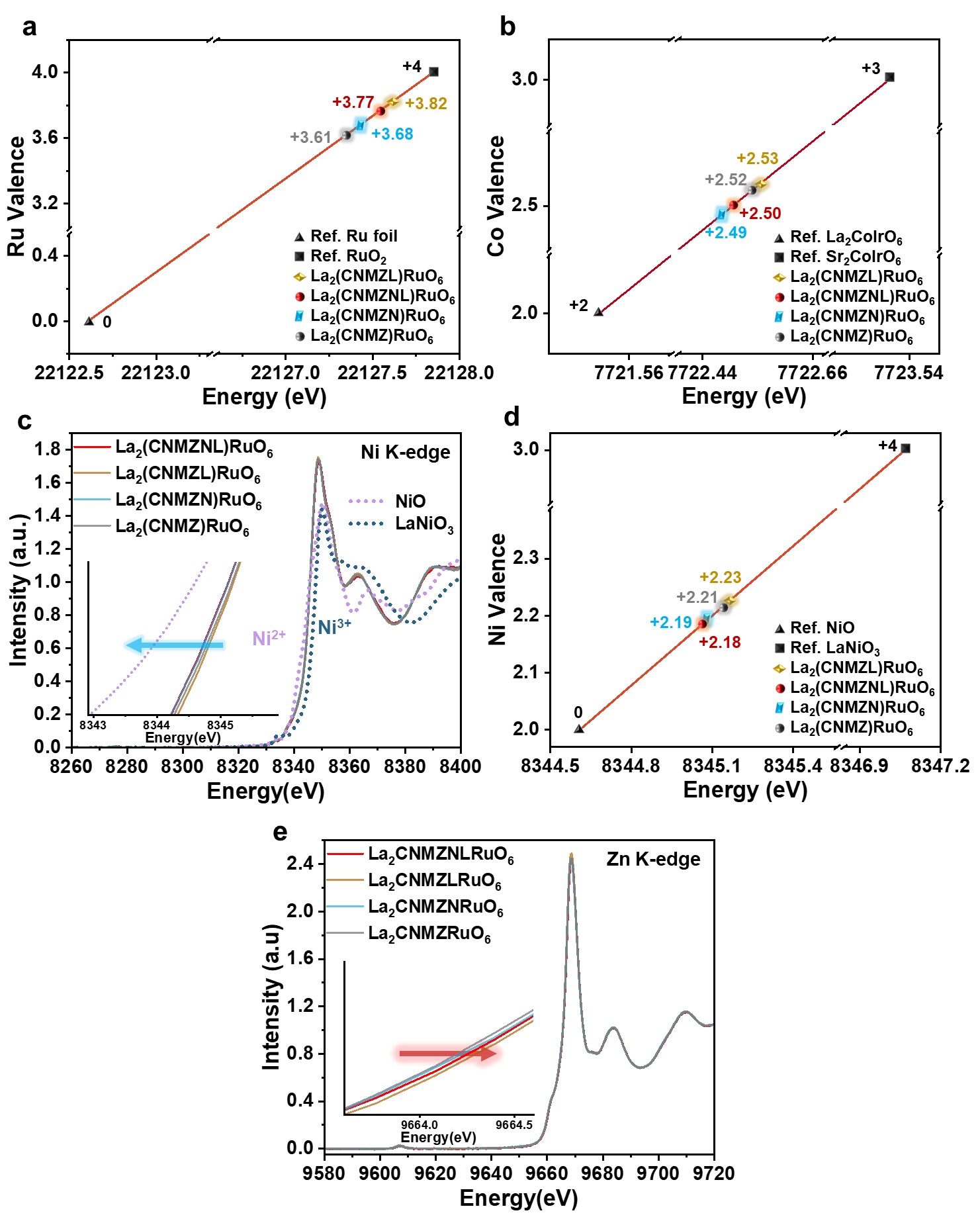


**Figure S10.** Calculated valence states of a) Ru K-edge and b) Co K-edge. XANES spectra for the c) Ni K-edge. Calculated valence states of d) Ni K-edge. XANES spectra for the e) Zn K-edge.


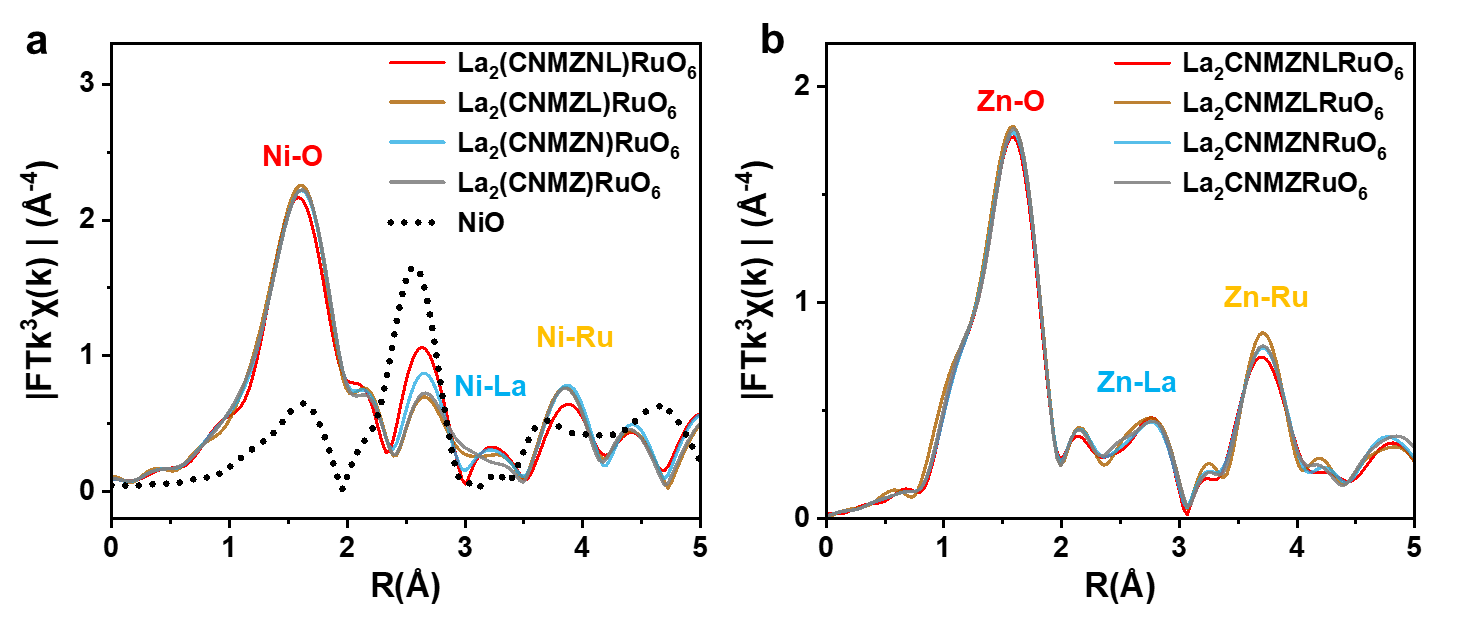


**Figure S11.** EXAFS spectra for c) Ni and d) Zn.


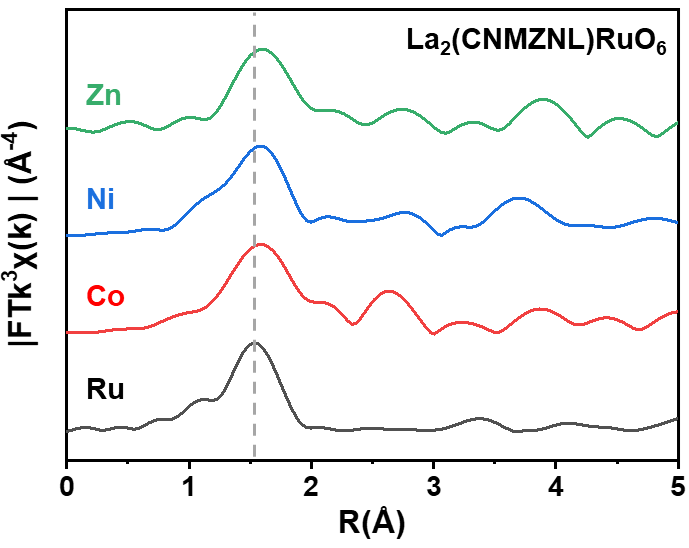


**Figure S12.** EXAFS spectra for Ru, Co, Ni and Zn in La_2_(CNMZNL)RuO_6_.


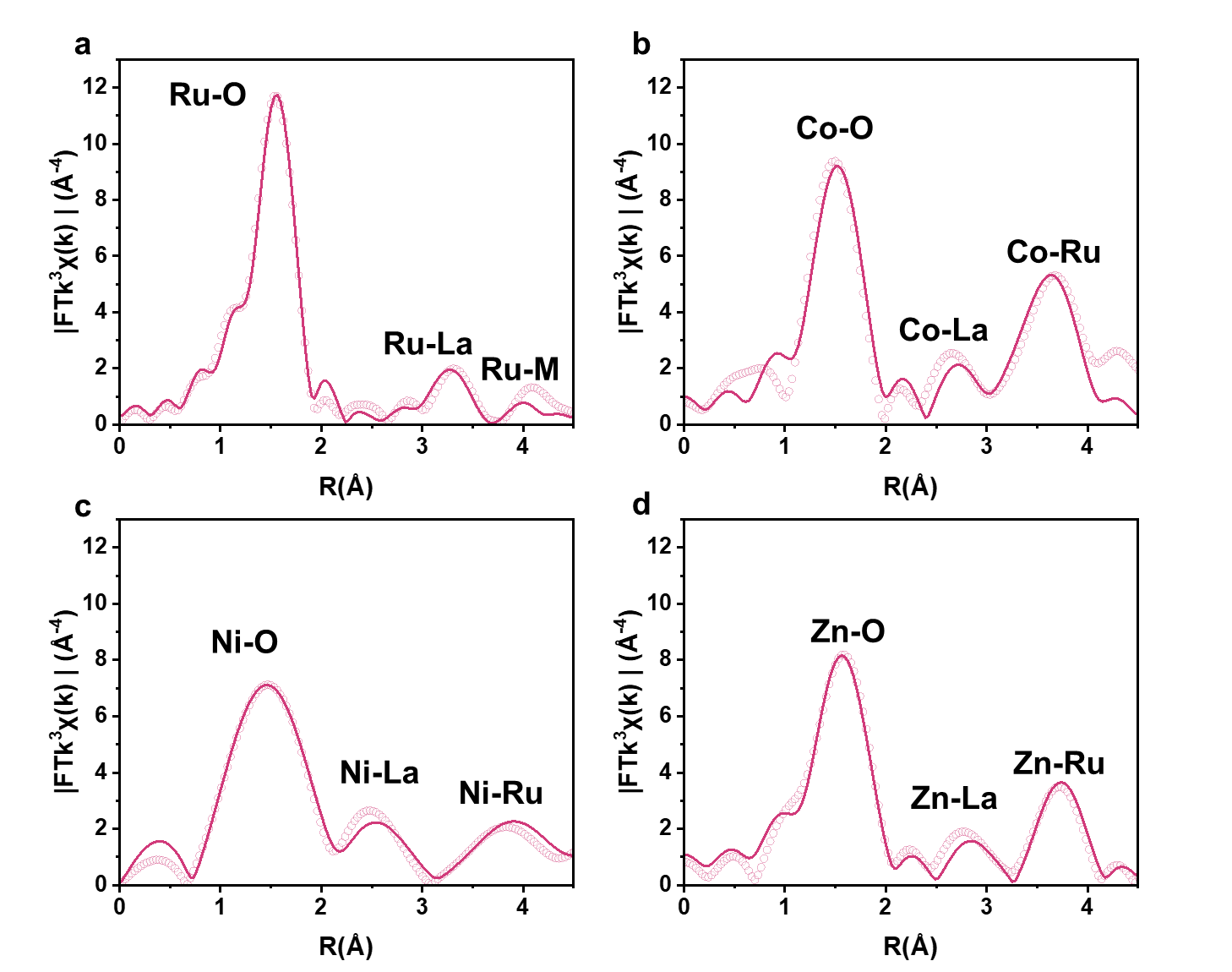


**Figure S13.** The EXAFS fitting XAFS spectra for Ru, Co, Ni and Zn in La_2_(CNMZNL)RuO_6_.


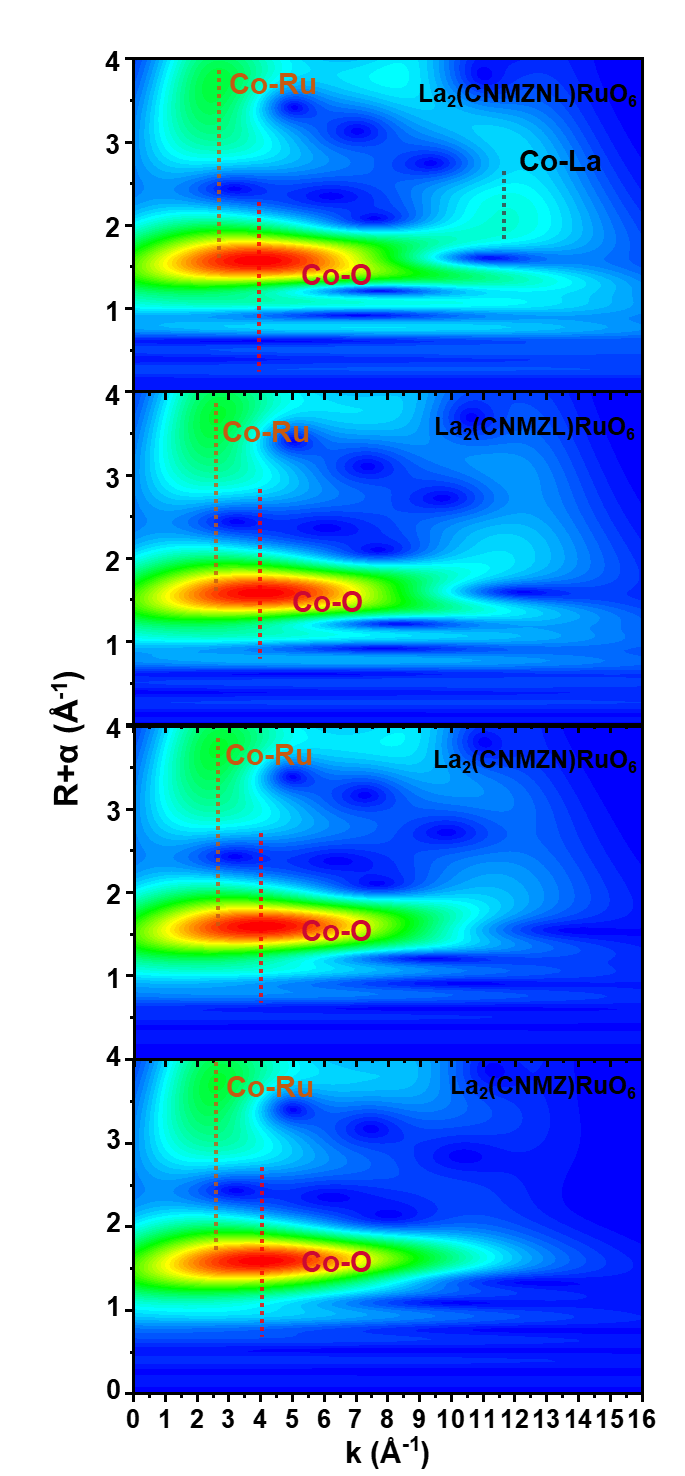


**Figure S14.** WT-EXAFS images of Co.


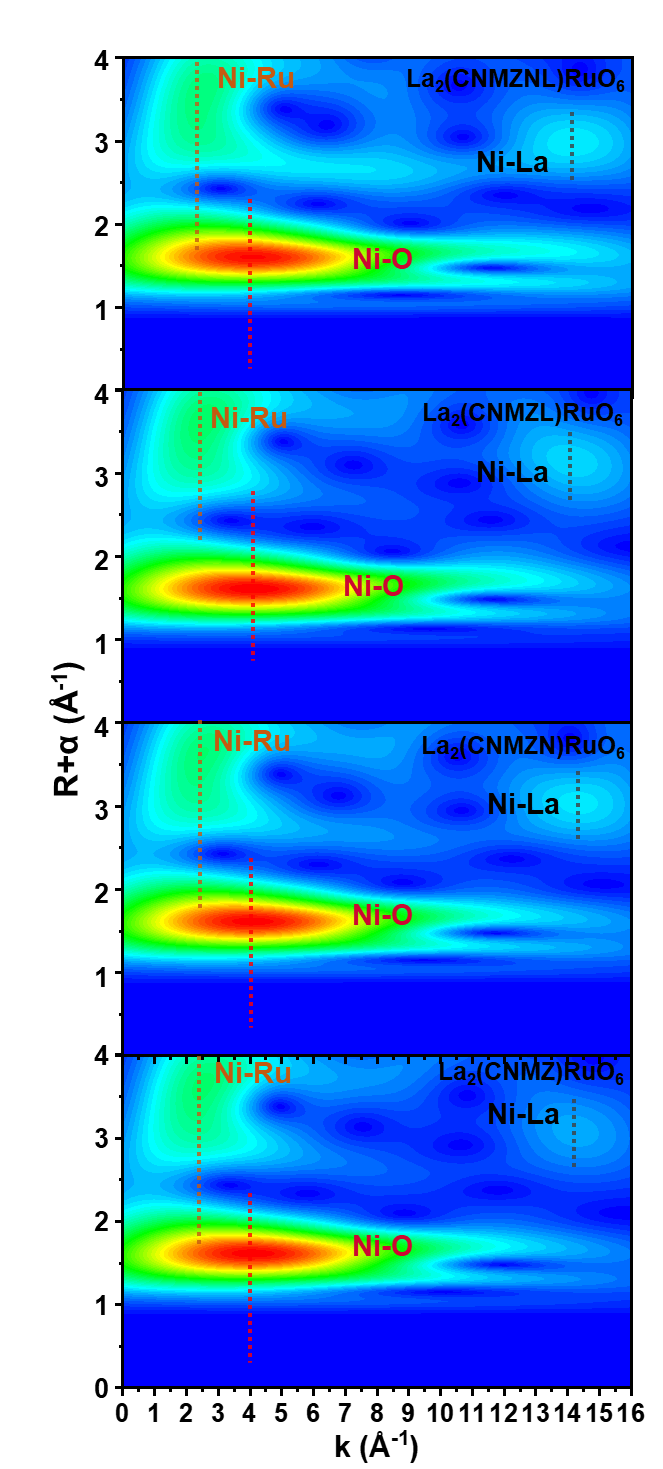


**Figure S15.** WT-EXAFS images of Ni.


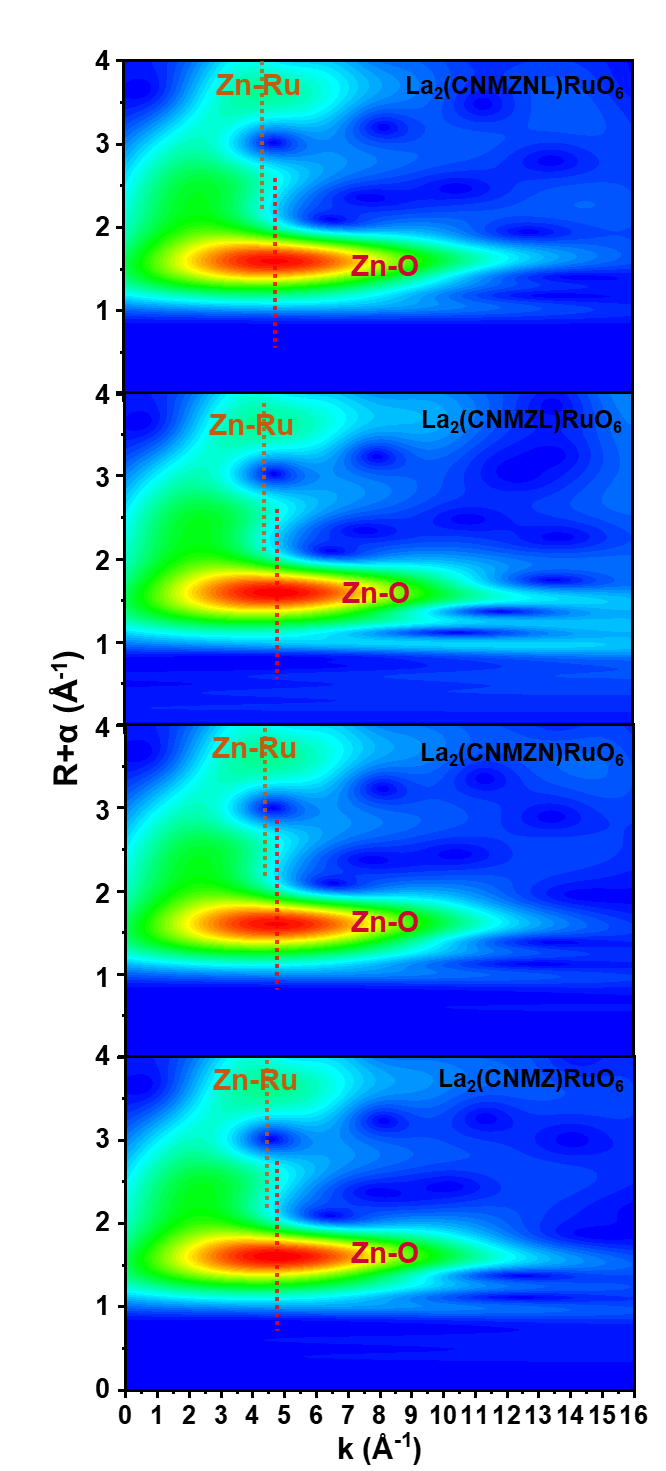


**Figure S16.** WT-EXAFS images of Zn.


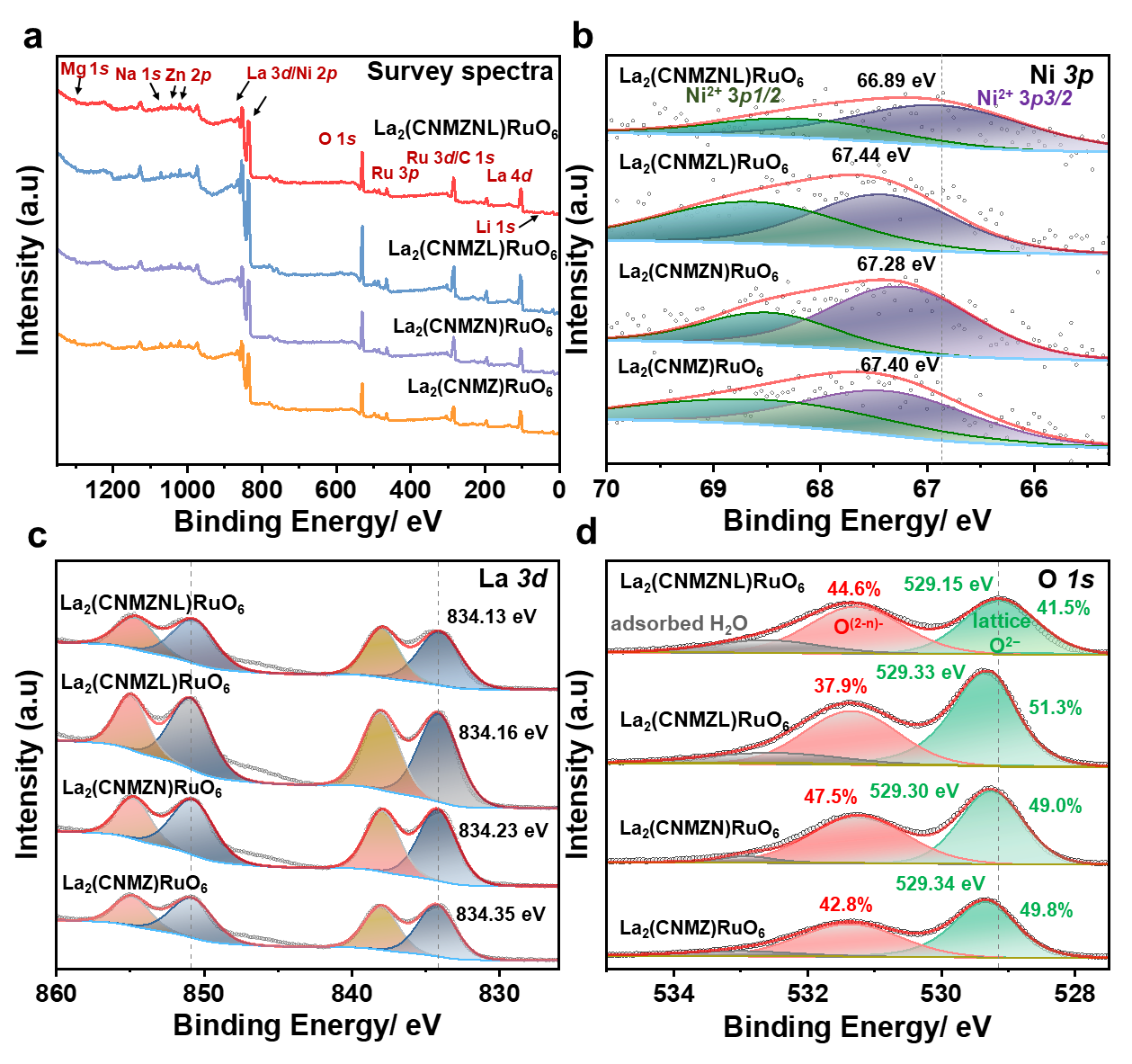


**Figure S17.** The electronic states on the electrode surface of La_2_(CNMZNL)RuO_6_, La_2_(CNMZL)RuO_6_, La_2_(CNMZN)RuO_6_, and La_2_(CNMZ)RuO_6_, catalysts by XPS. a) Total survey. b) High-resolution of XPS spectra for Ni 3*p*. c) High-resolution of XPS spectra for La 3*d*. d) High-resolution of XPS spectra for O 1*s*.


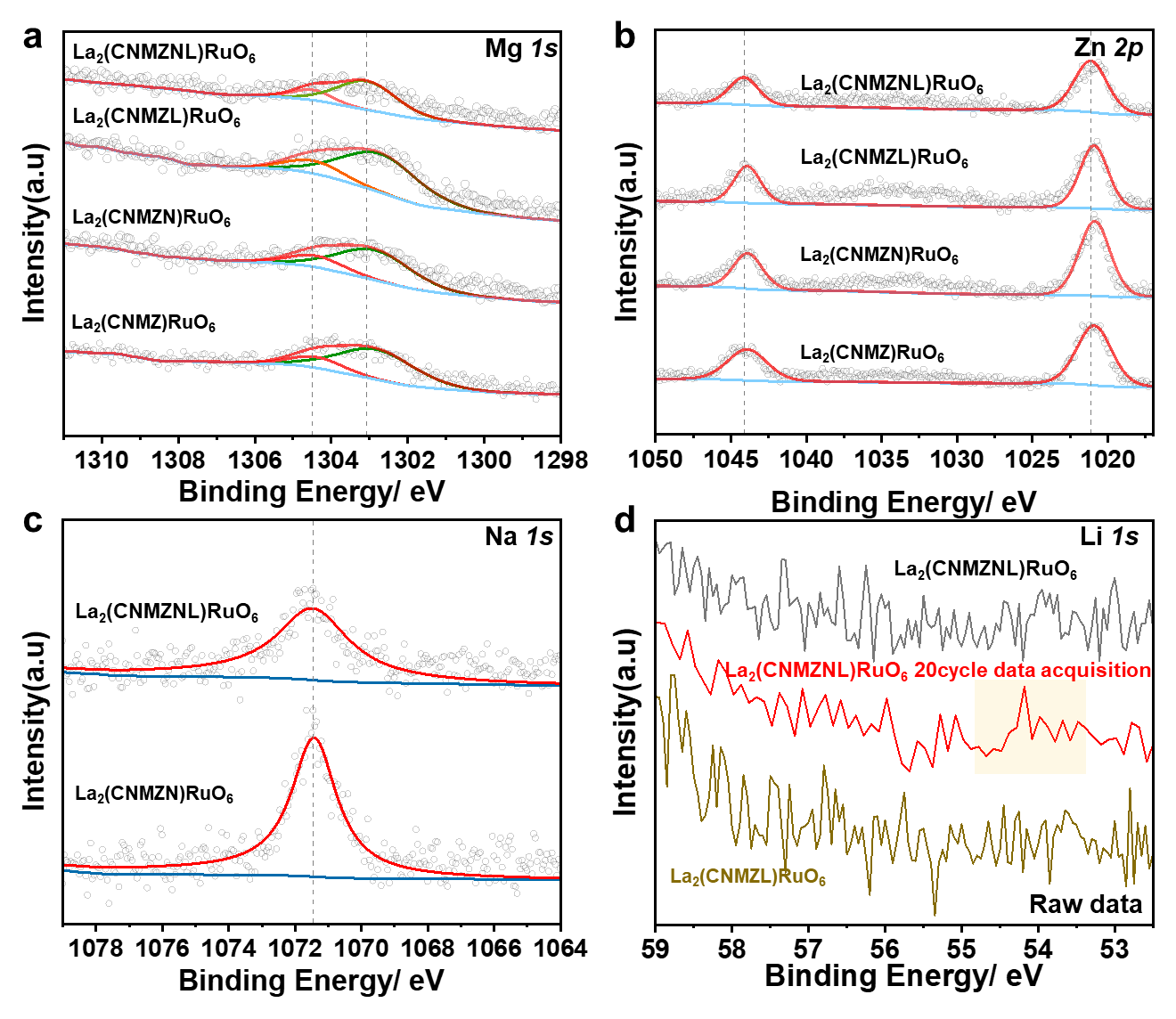


**Figure S18.** The electronic states on the electrode surface of La_2_(CNMZNL)RuO_6_, La_2_(CNMZL)RuO_6_, La_2_(CNMZN)RuO_6_, and La_2_(CNMZ)RuO_6_, catalysts by XPS. a-d) High-resolution of XPS spectra for a) Mg *1s*, b) Zn *2p*, c) Na *1s* and d) Li *1s*, respectively.

*Note:* *We conducted a 20-cycle superposition of XPS spectroscopic data on Li 1s to address the issue of low effective signal.* *To obtain Li 1s spectra with acceptable signal-to-noise ratios, we have taken care to perform integrations of sufficiently long duration. However, the noise content remains high. However, the Li 1s signal remains insignificant due to the low Li content in the high-entropy system, as reported in the literature.^[S1]^* *It is also pertinent to note that the Li 1s sensitivity factor is the lowest of all elements that can be detected by XPS.^[S2]^*

**
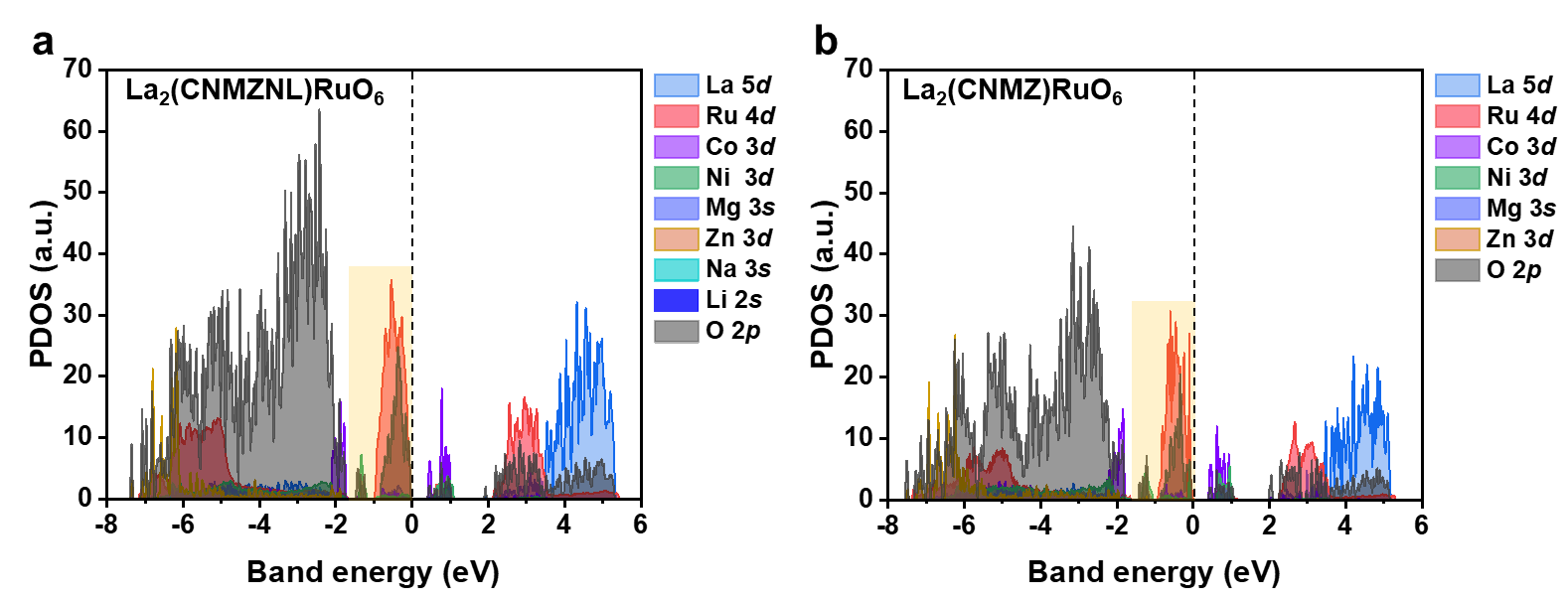
**

**Figure S19.** The PDOS within all high entropy elements of a) La_2_(CNMZNL)RuO_6_ and b) La_2_(CNMZ)RuO_6_.


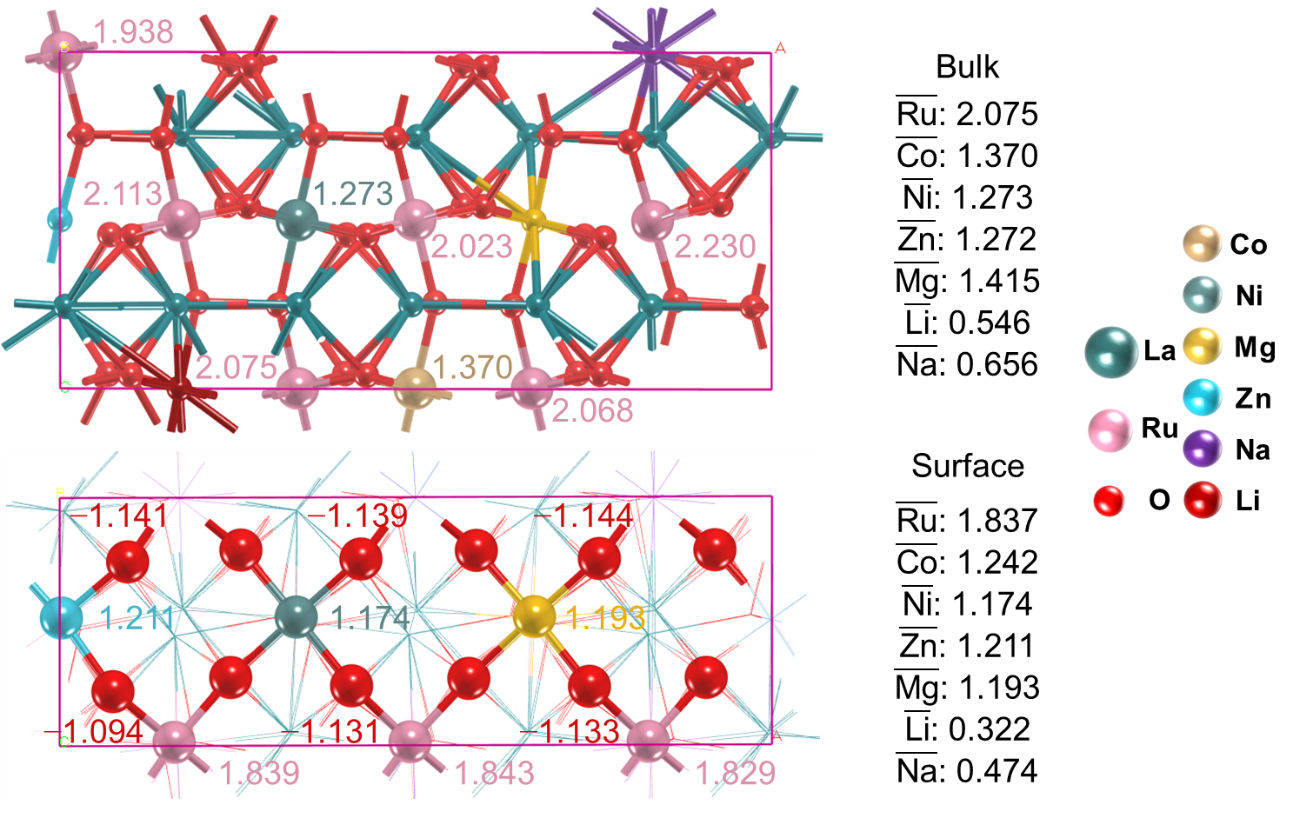


**Figure S20.** Average Bader charge of key metal elements (Ru, Co, Ni, Zn, Mg, Li, and Na) in the bulk and surface of the HEDP catalyst.

*Note:* *Bader charge analysis reveals a decrease in the surface valence state of metals compared to their bulk valance state, explaining why the oxidation state at the surface is much lower than that in the bulk. This is easy to understand because* *the disruption of the atomic bonding at the surface leads to their coordination unsaturation compared to the atom in the bulk, the surface oxygen has the higher activity, i.e., it receives the fewer electrons from the metals. Accordingly, the surface coordinated metals lose a small amount of electrons and exhibit lower oxidation states than those in the bulk.*


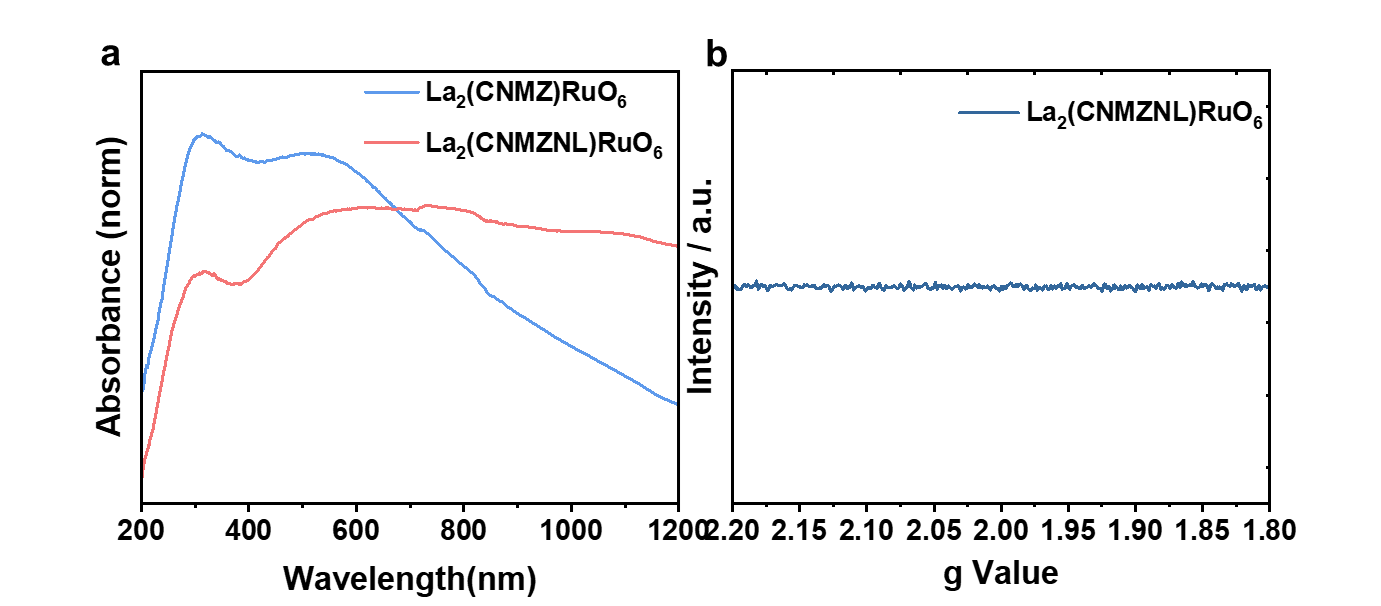


**Figure S21.** a) UV-visible spectra of La_2_(CNMZ)RuO_6_ and La_2_(CNMZNL)RuO_6_. b) EPR spectra of La_2_(CNMZ)RuO_6_ and La_2_(CNMZNL)RuO_6_.

**Figure S22.** The LSV curves of Ru-based double perovskites La_2_MRuO_6_, La_2_(CN)RuO_6_ and La_2_(CNM)RuO_6_ (M=Mn, Cu, Fe, Co, Ni, Mg, Li) .

**
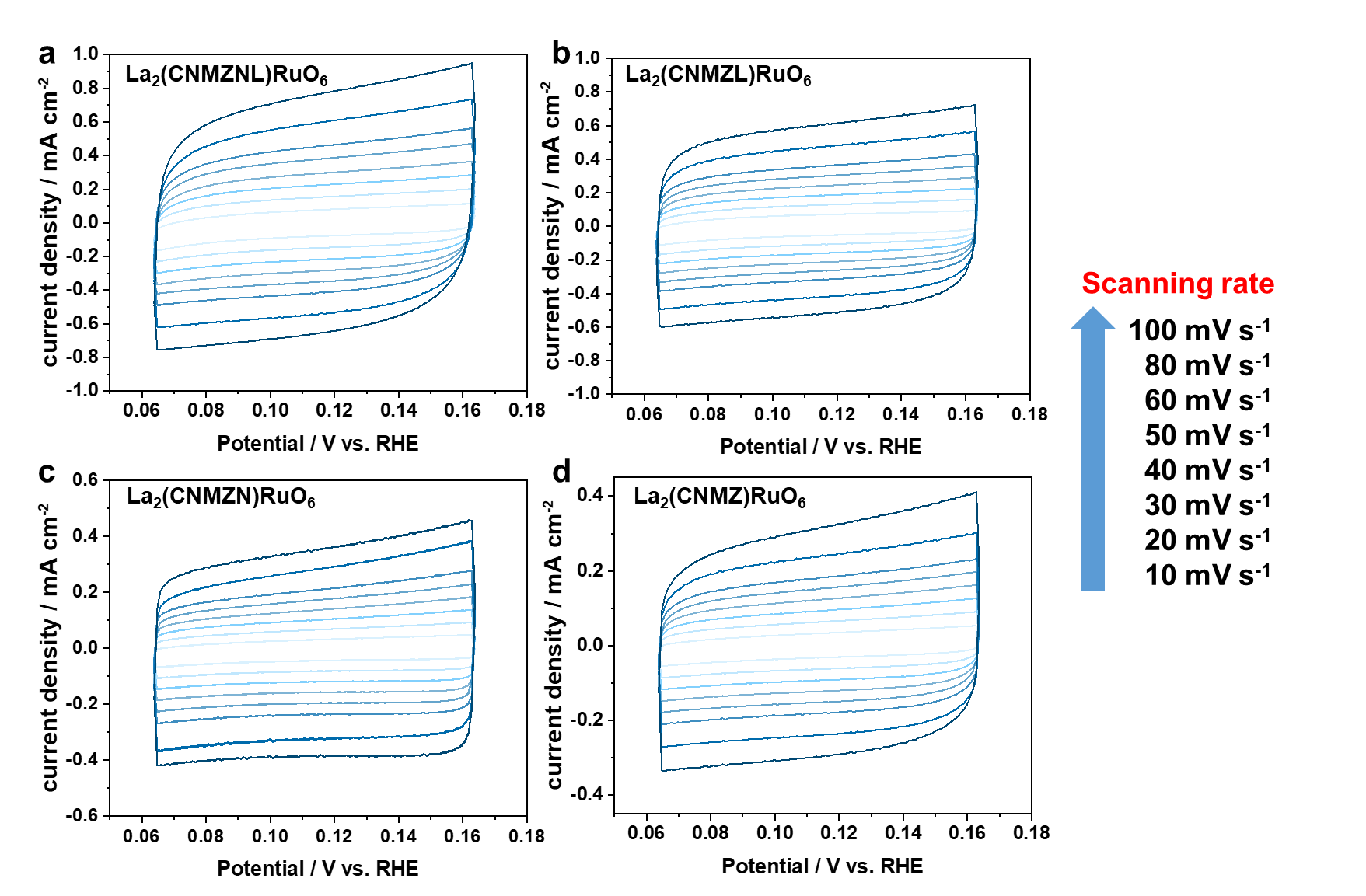
**

**Figure S23.** The CV curves of a) La_2_(CNMZNL)RuO_6_, b) La_2_(CNMZL)RuO_6_, c) La_2_(CNMZN)RuO_6_, d) La_2_(CNMZ)RuO_6_ samples with different rates from 10 to 100 mV s^-1^.


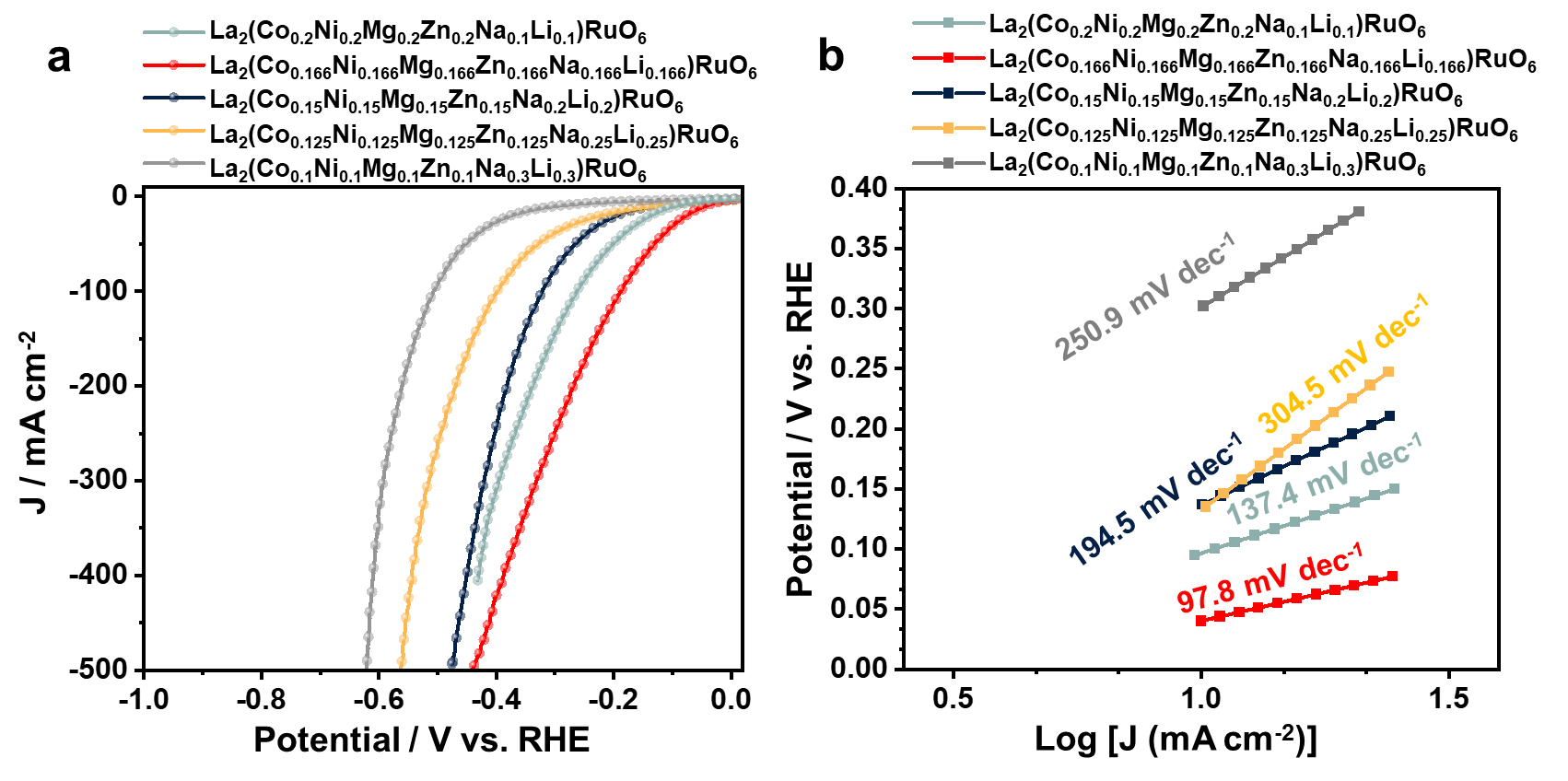


**Figure S24.** The LSV curves and Tafel slopes of different high-entropy perovskite with AM^+^: La_2_(Co_0.2_Ni_0.2_Mg_0.2_Zn_0.2_Na_0.1_Li_0.1_)RuO_6_, La_2_(Co_0.166_Ni_0.166_Mg_0.166_Zn_0.166_Na_0.166_Li_0.166_)RuO_6_, La_2_(Co_0.15_Ni_0.15_Mg_0.15_Zn_0.15_Na_0.2_Li_0.2_)RuO_6_, La_2_(Co_0.125_Ni_0.125_Mg_0.125_Zn_0.125_Na_0.25_Li_0.25_)RuO_6_ and La_2_(Co_0.1_Ni_0.1_Mg_0.1_Zn_0.3_Na_0.1_Li_0.3_)RuO_6_.


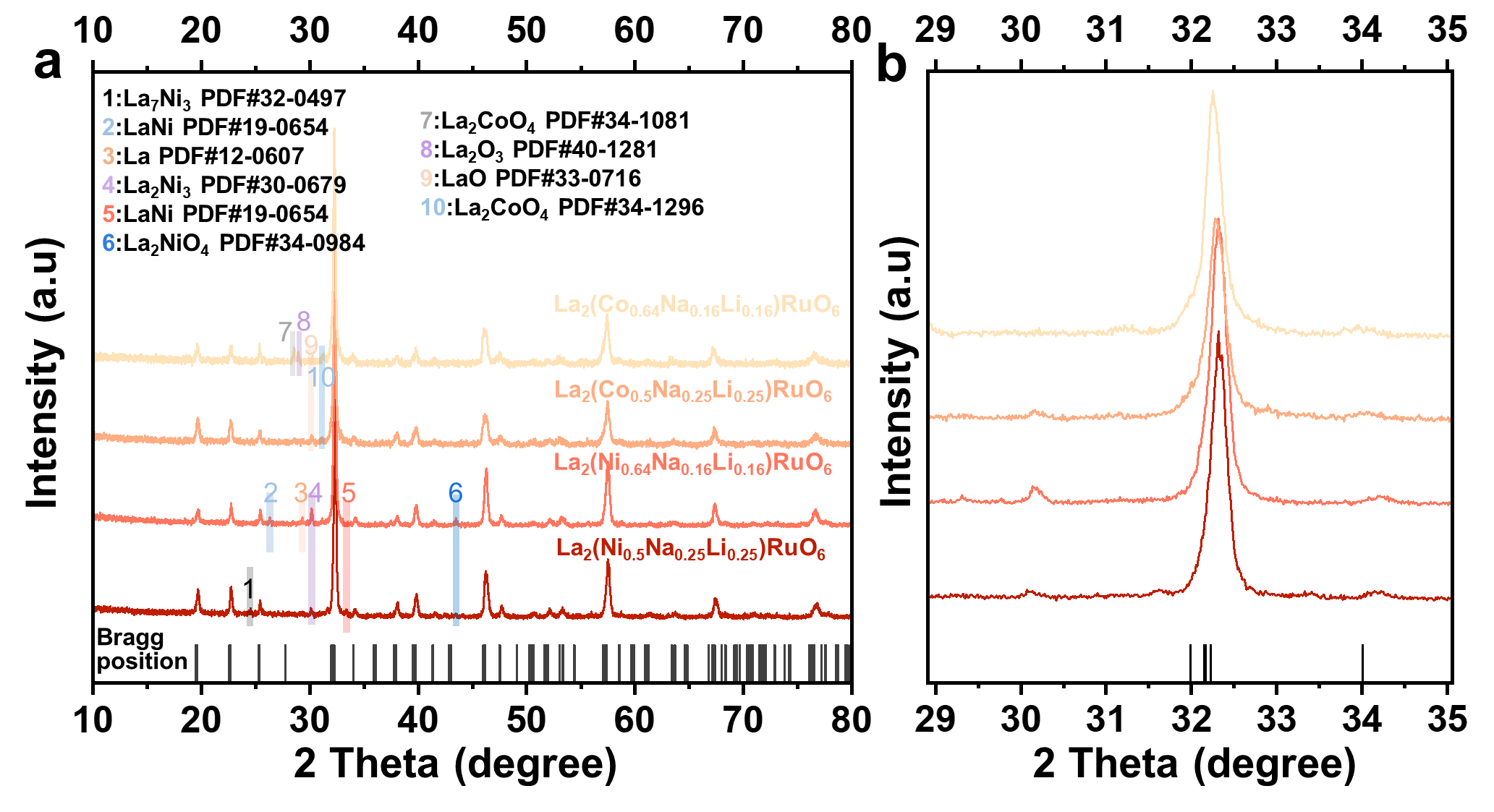


**Figure S25.** The XRD image of La_2_NiRuO_6_ and La_2_CoRuO_6_ with different content of AM^+^ for La_2_(Ni_0.5_Na_0.25_Li_0.25_)RuO_6_, La_2_(Ni_0.68_Na_0.16_Li_0.16_)RuO_6_ and La_2_(Co_0.5_Na_0.25_Li_0.25_)RuO_6_, La_2_(Co_0.68_Na_0.16_Li_0.1__6_)RuO_6_.

*Note: To evaluate the applicability of AM^+^ addition in enhancing the catalytic performance of double perovskites, we prepared the above-mentioned samples. It is evident that a little miscellaneous phases are present. However, with increasing AM^+^ content, the main peak at approximately 32.4° tends to shift leftward, possibly due to lattice stress alteration resulting from mismatch after AM^+^ introduction.^[S3]^ Therefore, we propose that the alkali metal is partially doped into the lattice and merits further investigation for its catalytic properties.*

**
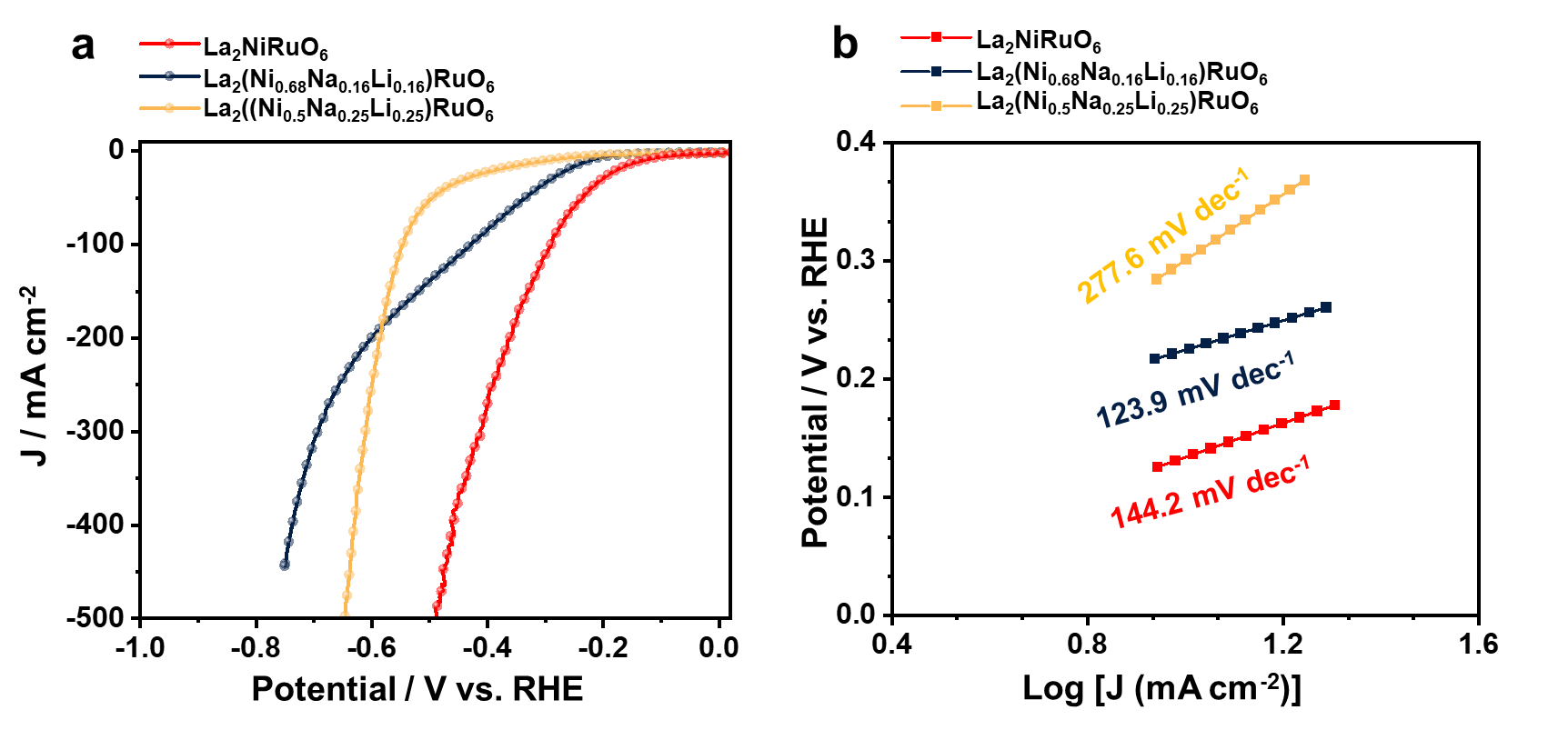
**

**Figure S26.** The LSV curves and Tafel slopes of double perovskite with/without AM^+^: La_2_(Ni_0.5_Na_0.25_Li_0.25_)RuO_6_, La_2_(Ni_0.68_Na_0.16_Li_0.16_)RuO_6_ and La_2_NiRuO_6_.


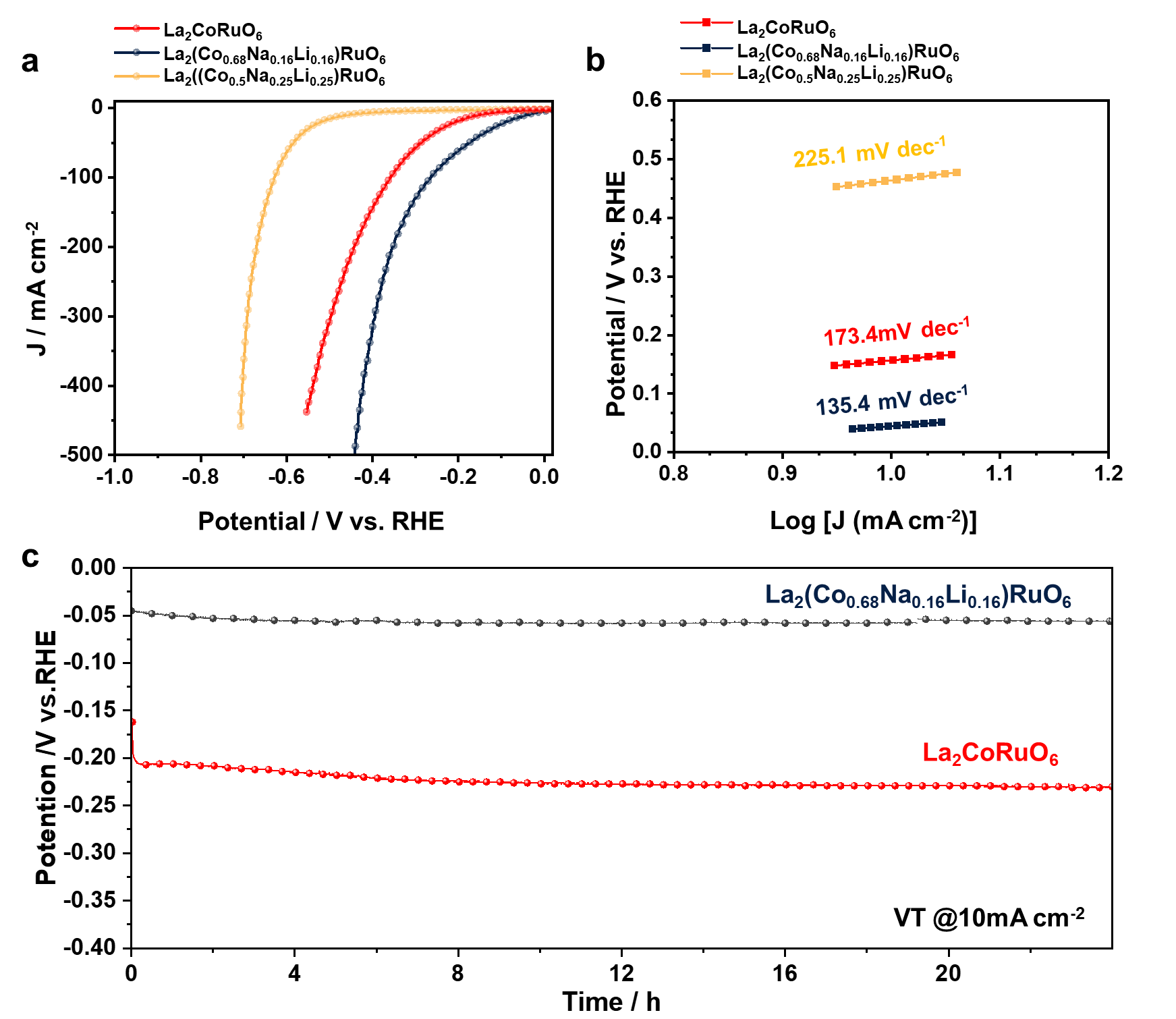


**Figure S27.** The LSV curves and Tafel slopes of double perovskite with/without AM^+^: La_2_(Co_0.5_Na_0.25_Li_0.25_)RuO_6_, La_2_(Co_0.68_Na_0.16_Li_0.16_)RuO_6_ and La_2_CoRuO_6_. The VT test under a constant current of 200 mA/cm^2^ of La_2_(Co_0.68_Na_0.16_Li_0.16_)RuO_6_ and La_2_CoRuO_6_. With the introduction of AM^+^, the stability of La_2_(Co_0.68_Na_0.16_Li_0.16_)RuO_6_ has improved significantly.

**
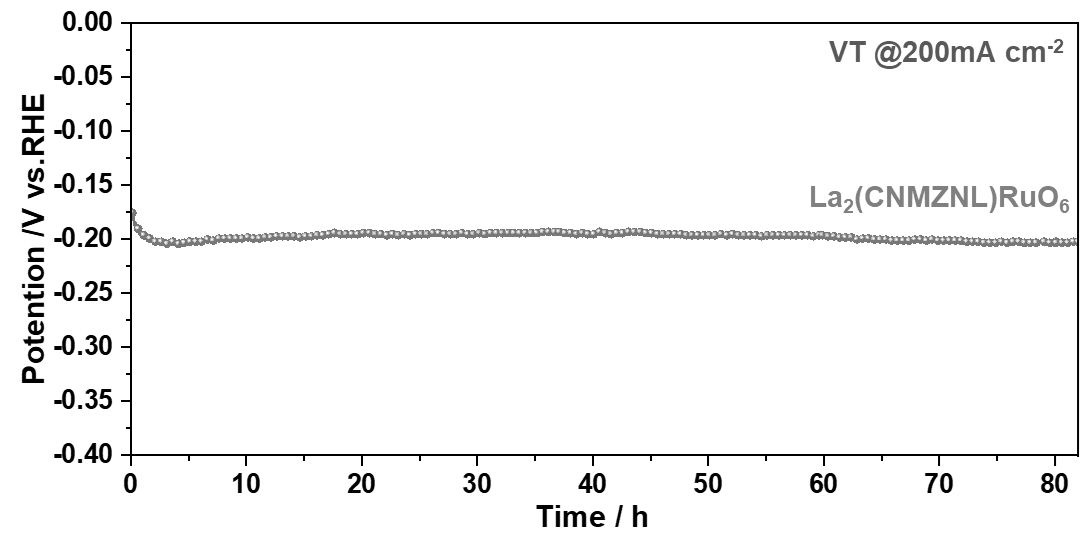
**

**Figure S28.** Stability measurement of the electrode under a constant current of 200 mA/cm^2^ for La_2_(CNMZNL)RuO_6_.


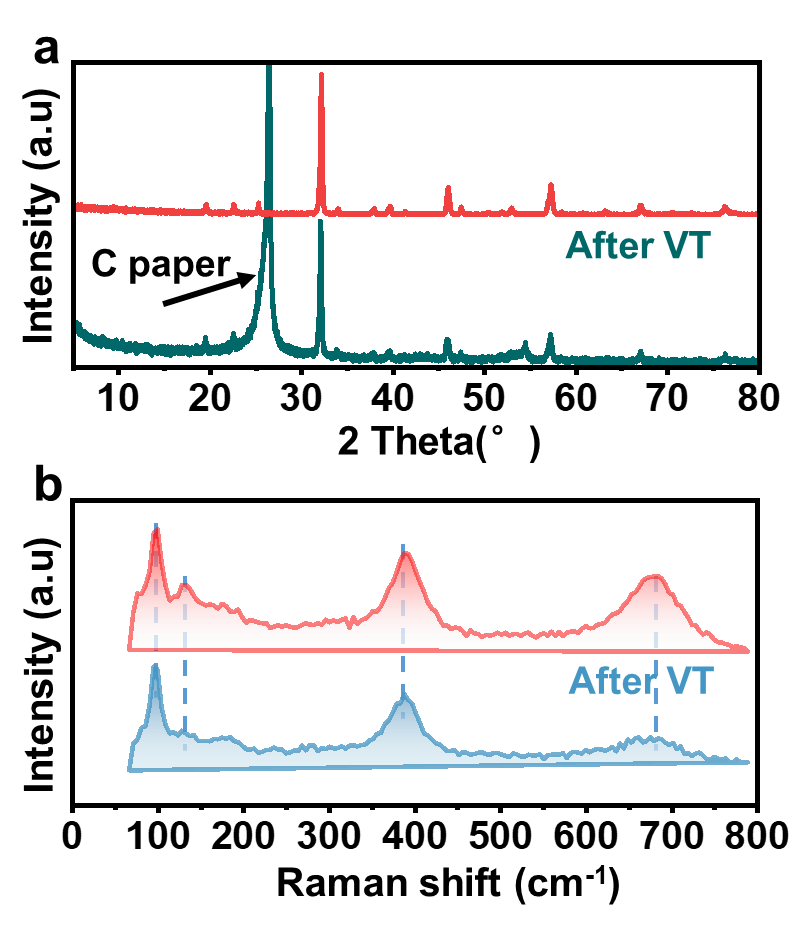


**Figure S29.** a) XRD for La_2_(CNMZNC)RuO_6_ after VT test. b) Raman spectrum for La_2_(CNMZNC)RuO_6_ after VT test.


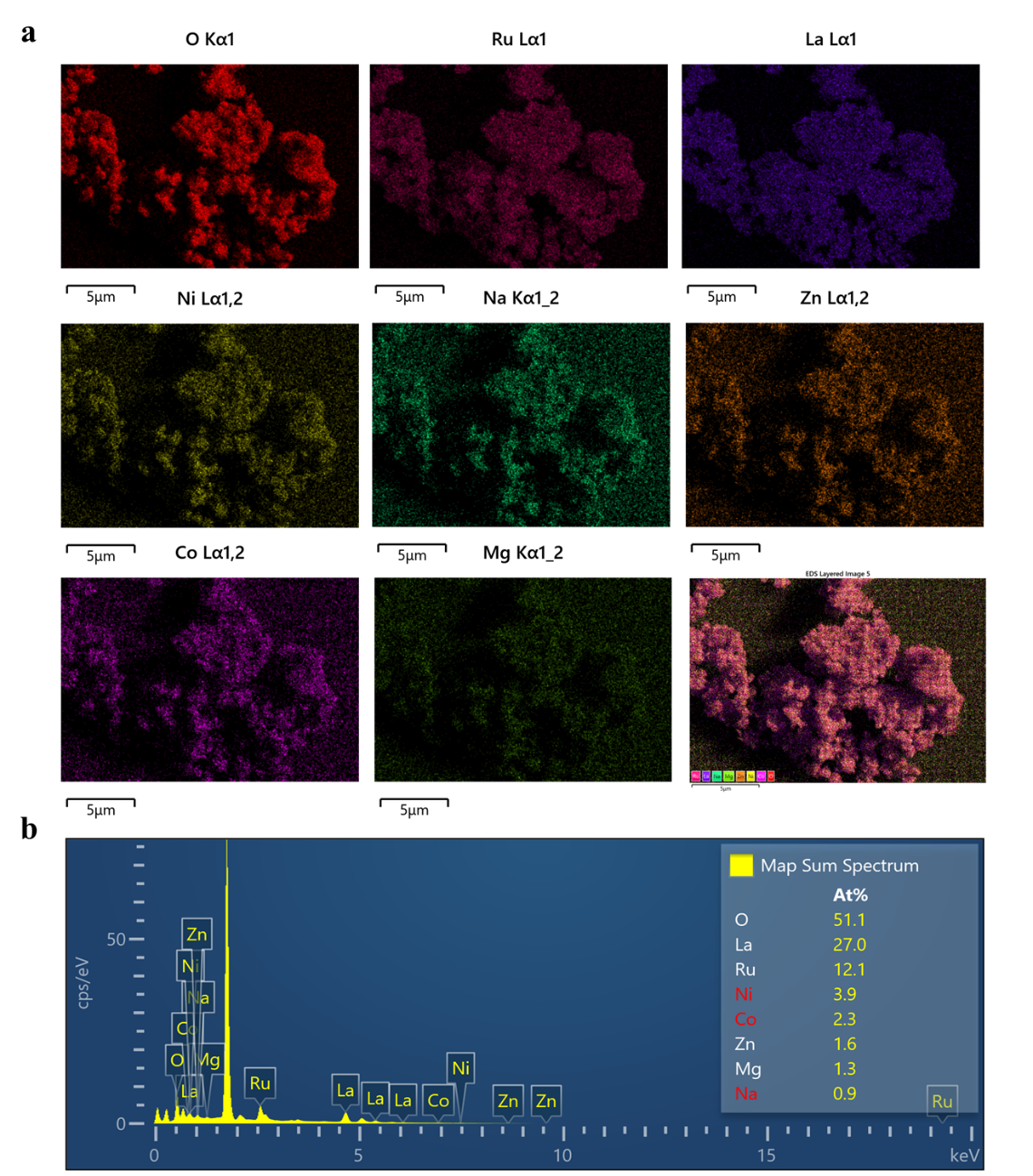


**Figure S30**. The SEM EDS-mapping of La_2_(CNMZNL)RuO_6_ after VT durability test.


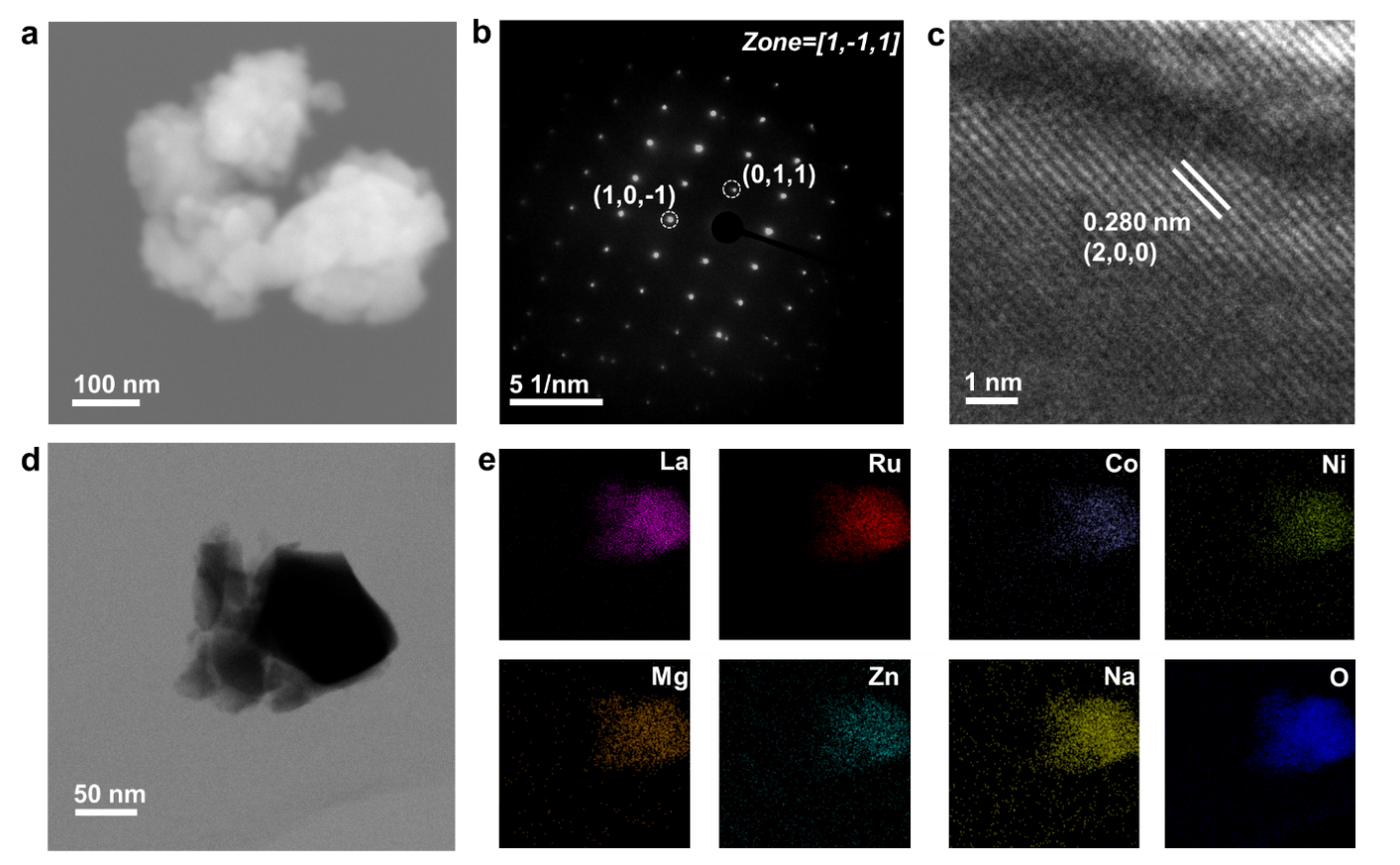


**Figure S31.** After stability test characterization of La_2_(CNMZNL)RuO_6_ catalysts. a) SEM image. b) SAED image. c) HR-TEM image. d-e) EDS-mapping image.


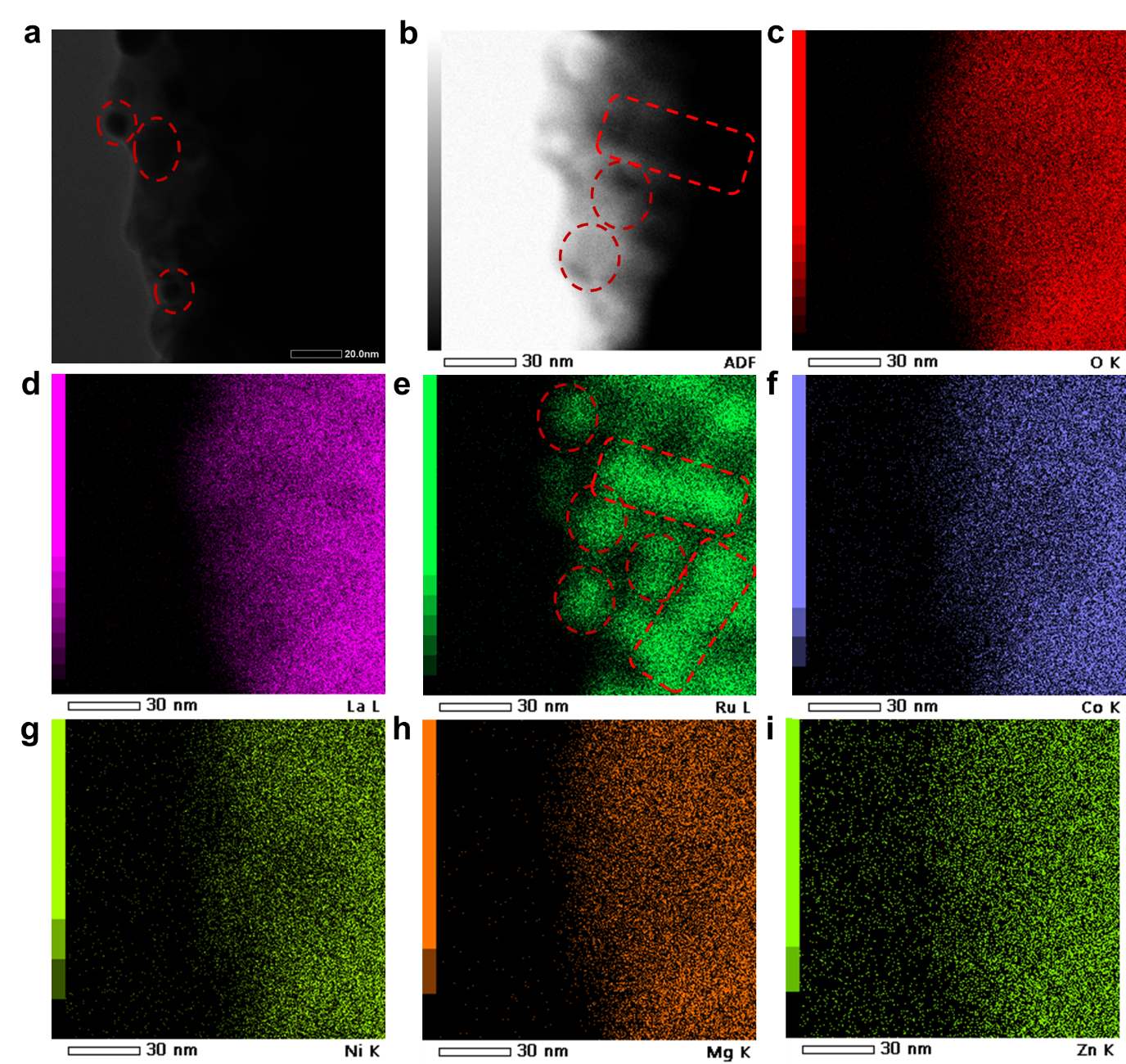


**Figure S32.** TEM of La_2_(CoNiMgZn)RuO_6_ catalysis after VT test. The red dotted line region is determined to be the generated Ru cluster.

**
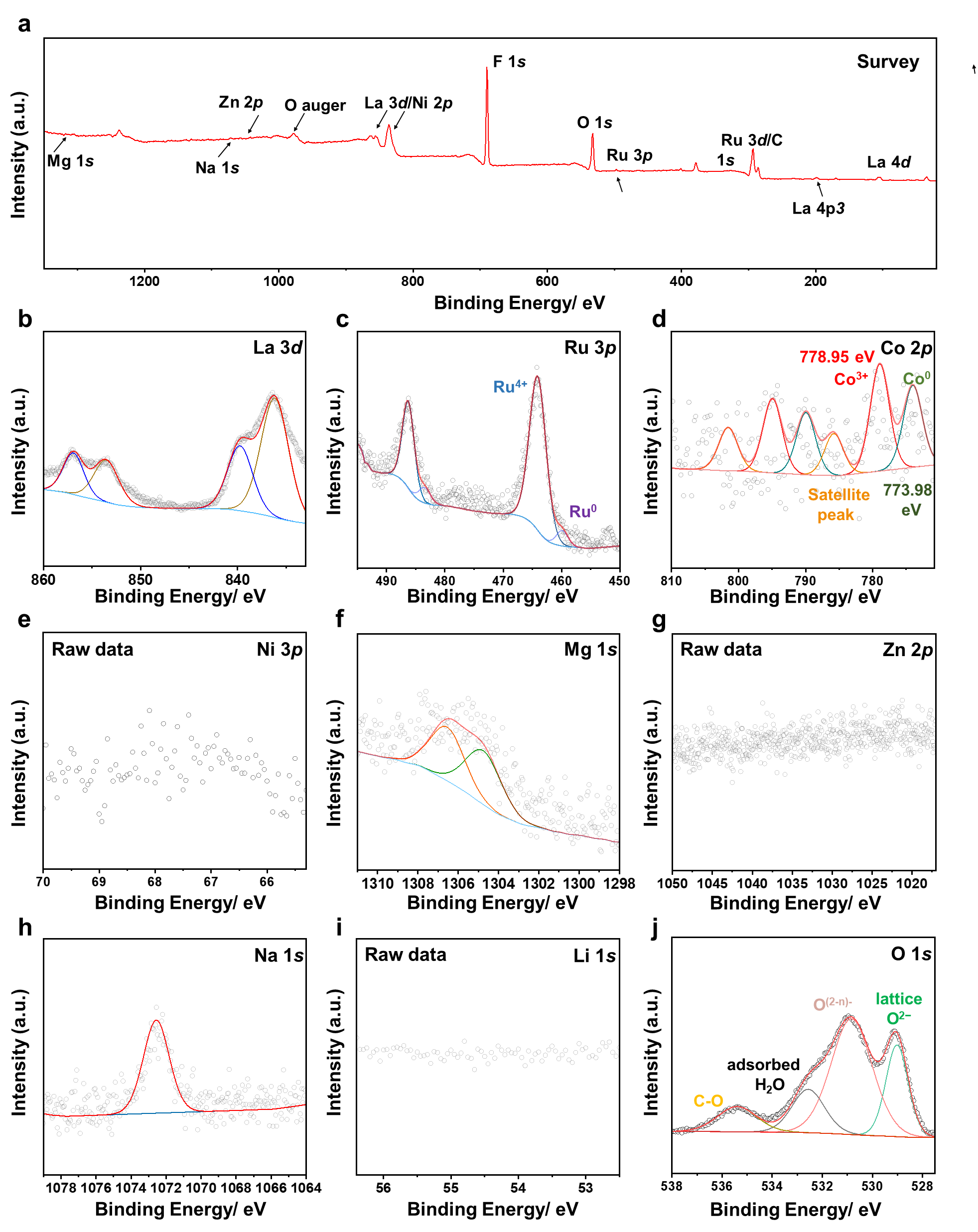
**

**Figure S33.** High-resolution of XPS spectra for La_2_(CoNiMgZn)RuO_6_ after VT test. a) Total survey. b) La 3*d*. c) Ru 3*p*. d) Co 2*p*. e) Ni 3*p*. f) Mg 1*s*. g) Zn 2*p*. h) Na 1*s*. i) Li 1*s*. j) O 1*s*.

*Note:* *Certain elements and orbitals may not be clearly discernible after VT test, resulting in significant noise signals.* *Discrimination between the different chemical states of Ni 3p, Zn 2p and Li 1s cannot be performed due to the insufficient signal-to noise ratio However, this does not imply that these elements are entirely absent from the surface, as confirmed by other characterizations including EDS and ICP.*


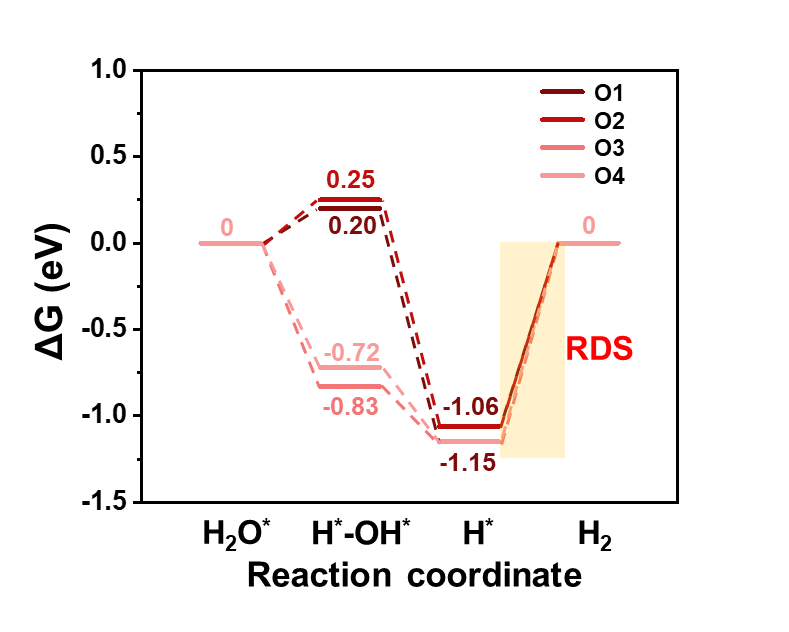


**Figure S34.** Alkaline HER pathway Gibbs free energy diagrams for La_2_(CNMZNL)RuO_6_ wherein where the dissociated H is adsorbed on O sites.

**Table S1.** The denotation of catalysts and the corresponding configuration entropy calculation.

| Composition of the samples | Denotation | Configuration entropy (R)^b^ |
| --- | --- | --- |
| La_2_(CoNiMgZnNaLi)RuO_6_^a^ | La_2_(CNMZNL)RuO_6_ | 1.27 |
| La_2_(CoNiMgZnNa)RuO_6_ | La_2_(CNMZN)RuO_6_ | 1.25 |
| La_2_(CoNiMgZnLi)RuO_6_ | La_2_(CNMZL)RuO_6_ | 1.25 |
| La_2_(CoNiMgZnMn)RuO_6_ | La_2_(CNMZM)RuO_6_ | 1.25 |
| La_2_(CoNiMgZnFe)RuO_6_ | La_2_(CNMZF)RuO_6_ | 1.25 |
| La_2_(CoNiMgZnCu)RuO_6_ | La_2_(CNMZC)RuO_6_ | 1.25 |
| La_2_(CoNiMgZn)RuO_6_ | La_2_(CNMZ)RuO_6_ | 1.23 |
| La_2_(CoNiMg)RuO_6_ | La_2_(CNM)RuO_6_ | 1.2 |
| La_2_(CoNi)RuO_6_ | La_2_(CN)RuO_6_ | 1.16 |

a: High entropy elements in the same proportion according to the nominal proportion of ingredients. Nominally, the addition of elements in parentheses is equal to the proportion of ruthenium.

b: Typically, the configurational entropy of a specific perovskite oxide system can be calculated using Equation (1)^[S4,S5]^:

$S_{\text{Configuration entropy}}=-\left\{ \left( \text{R}\sum_{\text{i=1}}^{\text{n}} \text{X}_{\text{i}}\text{×Ln}\text{X}_{\text{i}} \right)_{\text{A}}+\left( R\sum_{\text{k=1}}^{\text{n}} X_{k}\times LnX_{k} \right)_{\text{ B}^{\text{''}}}+\left( R\sum_{l=1}^{n} X_{l}\times LnX_{l} \right)_{\text{ B}^{\text{''}}}+\left( \text{R}\sum_{\text{m=1}}^{\text{n}} \text{X}_{\text{m}}\text{×Ln}\text{X}_{\text{m}} \right)_{\text{O-anion}} \right\}$ (1)

where *n* denotes the number of components in the A-cation-site, B'-cation-site and B''-cation-site and O-anion-site within our material system, *X_i_*, *X_k_*, *X_l_* and *X_m_*, represent the mole fraction of each respective A-cation-site, B'-cation-site and B''-cation-site and O-anion-site element, respectively, and *R* is the ideal gas constant (8.314 J mol^−1^ K^−1^). In our high entropy double perovskite platform, various configurational entropy parameters of the sample have been calculated and updated in the supplementary information.

**Table S2.** The refined lattice parameters and reliability factors of the sample changes with configuration entropy.

| Samples | Lattice paramaters | | | | | | R_wp_ (%) | GOF |
| --- | --- | --- | --- | --- | --- | --- | --- | --- |
|  | a (Å) | b (Å) | c (Å) | α (º) | β (º) | γ (º) |  |  |
| La_2_(CNMZNL)RuO_6_ | 5.5752 | 5.6242 | 7.8885 | 90.0000 | 90.0197 | 90.0000 | 12.26 | 1.05 |
| La_2_(CNMZN)RuO_6_ | 5.5746 | 5.6303 | 7.8908 | 90.0000 | 89.9490 | 90.0000 | 5.12 | 1.15 |
| La_2_(CNMZL)RuO_6_ | 5.5749 | 5.6139 | 7.8857 | 90.0000 | 90.0320 | 90.0000 | 5.28 | 1.10 |
| La_2_(CNMZ)RuO_6_ | 5.5767 | 5.6219 | 7.8923 | 90.0000 | 90.0172 | 90.0000 | 12.98 | 1.10 |
| La_2_(CNM)RuO_6_ | 5.5744 | 5.6130 | 7.8875 | 90.0000 | 89.9866 | 90.0000 | 5.41 | 1.1 |
| La_2_(CN)RuO_6_ | 5.5678 | 5.6184 | 7.8807 | 90.0000 | 89.9916 | 90.0000 | 4.83 | 1.1 |

**Table S3.** Chemical composition of La_2_(CNMZNL)RuO_6_ HEDP by the ICP-OES and EDS-mapping.

| Characterization measure | La (%) | Ru (%) | Co (%) | Ni (%) | Mg (%) | Zn (%) | Na (%) | Li (%) | O (%) |
| --- | --- | --- | --- | --- | --- | --- | --- | --- | --- |
| ICP-OES | 50.4 | 26.7 | 4.6 | 4.5 | 4.2 | 4.2 | 0.4 | 5 | / |
| EDS | 22.4 | 10.4 | 1.4 | 1.9 | 1.9 | 2.1 | 0.6 | / | 59.4 |

*Note: In fact, in much of the existing literature on high-entropy platforms, there is a lack of clear and consistent quantitative descriptions of Na, Li, and K content, mainly due to challenges in accurate characterization of these elements.^[S6-S9]^ Moreover, this challenge can also be attributed to the typically low alkali metal content in high-entropy compounds. Conventional quantitative and semi-quantitative characterization techniques such as EDS, XPS, and ICP may therefore be inadequate for accurately detecting these elements in trace amounts.*

*For instance, ICP-OES operates by nebulizing the sample solution into a high-temperature plasma created by subjecting argon gas to a high-frequency field.^[S10]^ Spectrochemical analysis methods like ICP-OES are often susceptible to errors caused by ionization interferences, particularly when there are changes in the ionization equilibrium state. Ionization interferences can significantly impact ICP-OES measurements, especially in the presence of easily ionizable elements such as Na, K, Rb, and Cs.^[S11]^*

*To achieve an equal distribution of alkali metals (Na, Li) alongside Co, Ni, Mg, and Zn in synthetic samples based on stoichiometric ratios, efforts have been made to ensure uniformity. However, alkali metals, especially sodium, are susceptible to volatilization during synthesis. Although a tube sealing technique has been employed to mitigate this issue, it is reasonable to expect that the alkali metal content may still be slightly lower compared to the other metallic elements in the samples. XRD refinement, EDS, ICP, and other characterization methods indicate that the proportions of Co, Ni, Mg, and Zn are nearly equal.*

**Table S4.** Average Bader charge of each element in HEDP catalyst and LaRuO_3_.

| Material | Element | Bader charge | Calculation model |
| --- | --- | --- | --- |
| HEDP catalyst | La | +2.114 \|e\| | 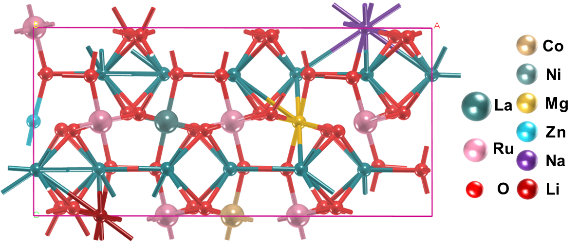 |
|  | Ru | +2.075 \|e\| |  |
|  | O | −1.232 \|e\| |  |
|  | Co | +1.370 \|e\| |  |
|  | Ni | +1.273 \|e\| |  |
|  | Mg | +1.415 \|e\| |  |
|  | Zn | +1.272 \|e\| |  |
|  | Li | +0.546 \|e\| |  |
|  | Na | +0.656 \|e\| |  |
| LaRuO_3_ | La | +2.210 \|e\| | 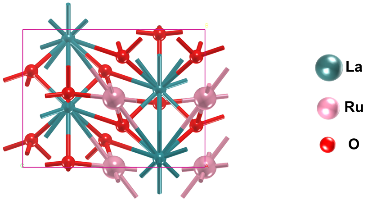 |
|  | Ru | +1.751 \|e\| |  |
|  | O | −1.980 \|e\| |  |

*Note: When doping the low valence state metals (e.g. Mg, Na, Li) to form HEDP catalyst, if oxygen remains in the −2 valence state and no oxygen vacancy is found, the charge balance of the system is very likely to be a problem. We have performed the Bader charge analysis of each element on the* *HEDP catalyst. Taking the pure LaRuO_3_ as the reference state, we can see that the introduction of the low valence state metals (e.g. Mg, Na, Li, Zn) significantly changes the charge of the Ru and O elements, causing the increase of Ru (from +1.751 |e| to +2.075 |e|) and O (from −1.980 |e| to −1.232 |e|) valance states. This suggests that the charge of the system can be balanced by the rearrangement of elemental electrons, reflecting the influence of electron factors on the performance of the HEDP catalyst.*

**Table S5.** Structural parameters of La_2_(CNMZNL)RuO_6_ catalyst derived from EXAFS fitting.

| Sample | Path | CN | σ^2^ | ΔE_0_ (eV) | R (Å) | R factor |
| --- | --- | --- | --- | --- | --- | --- |
| La_2_(CNMZNL)RuO_6_ | Ru-O | 6 | 0.0035 | 0.358 | 2.0089 | 0.018 |
|  | Ru-La1 | 2 | 0.0069 | 0.358 | 3.4936 |  |
|  | Ru-La2 | 6 | 0.0052 | 0.358 | 3.9632 |  |
|  | Ru-M | 4.97 | 0.0025 | 0.358. | 4.0738 |  |
|  | Co-O | 6 | 0.0048 | -7.941 | 2.0098 | 0.019 |
|  | Co-La1 | 2 | 0.0092 | -7.941 | 3.1138 |  |
|  | Co-La2 | 6 | 0.0192 | -7.941 | 3.3134 |  |
|  | Co-Ru | 10.81 | 0.0089 | -7.941 | 3.9450 |  |
|  | Ni-O | 6 | 0.0001 | -8.730 | 2.0133 | 0.015 |
|  | Ni-La1 | 2 | 0.0020 | -8.730 | 3.2586 |  |
|  | Ni-La2 | 6 | 0.0020 | -8.730 | 3.6374 |  |
|  | Ni-Ru | 6 | 0.0378 | -8.730 | 3.8149 |  |
|  | Zn-O | 6 | 0.0071 | -8.217 | 2.0805 | 0.017 |
|  | Zn-La | 2 | 0.0093 | -8.217 | 3.2704 |  |
|  | Zn-La | 6 | 0.0387 | -8.217 | 3.4370 |  |
|  | Zn-Ru | 13 | 0.0150 | -8.217 | 3.9974 |  |

**Table S6.** Summary of recently reported representative perovskite and high entropy HER electrocatalysts in alkaline electrolytes.

| Catalysts | Tafel slope  (mV dec^-1^) | *η_10_* (mV) | *η_100_* (mV)^a^ | *η_200_* (mV)^a^ | Reference |
| --- | --- | --- | --- | --- | --- |
| La_2_(CNMZNL)RuO_6_ | 97.8 | 40.7 | 158.2 | 205 | This work |
| LSTN (La_0.4_Sr_0.4_Ti_0.9_Ni_0.1_O_3-&_) @C_3_N_4_ | 68 | 225 | / | / | [S12] |
| LnBaCo_2_O_5+δ_ | 64.4 | 156 | ~252 | ~305 | [S13] |
| Pr_0.5_BSCF | 45 | 237 | ~305 | / | [S14] |
| La_0.5_(Ba_0.4_Sr_0.4_Ca_0.2_)_0.5_Co_0.8_Fe_0.2_O_3–δ_ (L-0.5)/rGO | 80 | 144 | ~182 | / | [S15] |
| La_0.4_Sr_0.4_Ti_0.9_O_3-δ_ (LST) | 97 | 420 | / | / | [S16] |
| PrBaCo_2_O_5-δ_ | 59.0 | 245 | / | / | [S17] |
| NC-CF-PSFN | 101.4 | 186 | / | / | [S18] |
| BaMoO_3_ | 110 | 336 | ~448 | / | [S19] |
| Co(OH_)2_/SFM-NF | 77 | 312 | / | / | [S20] |
| PBCO_5.8_ | 60 | 240 | / | / | [S21] |
| SFMON-450 | 138 | 251 | ~382 | ~422 | [S22] |
| Ce doping LaCoO_3_ | 144 | 305 | / | / | [S23] |
| Vo-LaCoO_3_ | 91 | N/A | / | / | [S24] |
| mC-Mo-850 | 55 | 145 | ~222 | ~272 | [S25] |
| LaCo_1-x_Pt_x_O_3-δ_ | 148 | 294 | / | / | [S26] |
| SrLaFe_1-x_Co_x_O_4-δ_ | 91 | 547 | / | / | [S27] |
| S-Nd_0.6_Sr_0.4_Co_0.6_Fe_0.3_Nb_0.1_O_3-δ_ | 133 | 470 | / | / | [S28] |
| CaSrFe_0.75_Co_0.75_Mn_0.5_O_6−δ_ (CSFCM) | N/A | 310 | / | / | [S29] |
| Sr_0.95_Co_0.7_Nb_0.1_Ni_0.2_O_3-δ_ (S2-400) | 80 | 208 | ~425 | ~448 | [S30] |
| LaCa_2_Fe_3_O_8_ | 143 | 400 | ~681 | / | [S31] |
| Sr_x_Ca_3−x_GaMn_2_O_8_ | 128 | 315 | / | / | [S32] |
| Pr_0.5_La_0.5_ | 33.8 | 210 | ~223 | ~231 | [S33] |
| SrT_i0.7_Ru_0.3_O_3-δ_ | 40 | 46 | ~120 | / | [S34] |
| PB_0.94_C-DSPH | 56.5 | 210 | ~283 | ~329 | [S35] |
| NdBaMn_2_O_5.5−δ_ | 87 | 290 | / | / | [S36] |
| 1P-HEA(PniCoCrFeAlW) | 32.6 | 70 | ~179 | ~216 | [S37] |
| FeCoNiCrCu | 62 | 84 | / | / | [S38] |
| La_0.5_Sr_0.5_CoO_3-x_ | 155 | / | ~330 | / | [S39] |

a: The data of other electrocatalysts were obtained using the GETDATA software. ^[S33]^

**Table S7.** EIS fitting results of catalysts including *R*_s_, *R*_ct_ and *R*_2_ values.

| Samples | *R*_s ­_(Ω) | *R*_ct ­­_(Ω) | *R*_2­_(Ω) |
| --- | --- | --- | --- |
| La_2_(CNMZNL)RuO_6_ | 2.66 | 0.96 | 9.59 |
| La_2_(CNMZN)RuO_6_ | 2.65 | 3.17 | 57.4 |
| La_2_(CNMZL)RuO_6_ | 2.56 | 1.48 | 10.52 |
| La_2_(CNMZ)RuO_6_ | 2.64 | 5.25 | 90.64 |

**Table S8.** Chemical composition of La_2_(CNMZNL)RuO_6_ HEDP by the ICP-OES after VT test duiring different durable time. ^a^

| Durable time | Constant current (mA/cm^2^) | La (μg/L) | Ru (μg/L) | Co (μg/L) | Ni (μg/L) | Mg (μg/L) | Zn (μg/L) | Li (μg/L) |
| --- | --- | --- | --- | --- | --- | --- | --- | --- |
| 24h | 10 | 5.7 | 10.6 | 0.4 | 2.8 | 10.1 | 8.2 | 8.9 |
| 82h | 10 | 12.8 | 14.2 | 0.7 | 3.4 | 11.5 | 9.4 | 10.0 |
| 82h | 200 | 2.4 | 46.1 | 2.0 | 9.0 | 11.3 | 25.1 | 12.7 |

a: Due to the unavoidable presence of Na in alkaline solutions of potassium hydroxide as a result of the manufacturing process, ICP characterization of Na is meaningless to be shown, as reported in the literature.

**References**

1. E. Lökçü, Ç. Toparli, M. Anik, *ACS Appl. Mater. Interfaces* **2020**, *12*, 23860.
2. K. N. Wood, G. Teeter, *ACS Appl. Energy Mater.* **2018**, *1*, 4493.
3. D. Guan, J. Zhong, H. Xu, Y.-C. Huang, Z. Hu, B. Chen, Y. Zhang, M. Ni, X. Xu, W. Zhou, Z. Shao, *Appl. Phys. Rev.* **2022**, *9*, 011422.
4. L. Tang, Y. Yang, H. Guo, Y. Wang, M. Wang, Z. Liu, G. Yang, X. Fu, Y. Luo, C. Jiang, Y. Zhao, Z. Shao, Y. Sun, *Adv. Funct. Mater.* **2022**,*32*, 2112157.
5. A. Sarkar, L. Velasco, D. Wang, Q. Wang, G. Talasila, L. de Biasi, C. Kübel, T. Brezesinski, S. S. Bhattacharya, H. Hahn, B. Breitung, *Nat. Commun.* **2018**, *9*, 3400.
6. C. Zhao, F. Ding, Y. Lu, L. Chen, Y.-S. Hu, *Angew. Chem. Int. Ed.,* **2020**, *59*, 264.
7. W. Jiang, T. Wang, H. Chen, X. Suo, J. Liang, W. Zhu, H. Li, S. Dai, *Nano Energy* **2021**, *79*, 105464.
8. T. Wang, H. Chen, Z. Yang, J. Liang, S. Dai, *J. Am. Chem. Soc.*, **2020**, *142*, 4550.
9. Y. Gu, A. Bao, X. Wang, Y. Chen, L. Dong, X. Liu, H. Pan, Y. Lie, X. Qi, *Nanoscale*, **2022**, *14*, 515.
10. M. Krachler, S. Van Winckel, M. Cardinale, B. Lynch, T. Murakami, *Microchem. J.*, **2012**, *105*, 9.
11. Y. Morishige, A. Kimura, *SEI. Tech. Rev.*, **2008**, *66*, 106.
12. U.A. Asif, T. Noor, E. Pervaiz, N. Iqbal, N. Zaman, *J. Alloys Compd.* **2023**, *939*, 168668.
13. Y. Dou, Y. Xie, X. Hao, T. Xia, Q. Li, J. Wang, L. Huo, H. Zhao, *Appl.Catal., B* **2021**, *297*, 120403.
14. X. Xu, Y. Chen, W. Zhou, Z. Zhu, C. Su, M. Liu, Z. Shao, *Adv. Mater.* **2016**, *28*, 6442.
15. B. Hua, M. Li, Y. Q. Zhang, Y. F. Sun, J. L. Luo, *Adv. Energy Mater.* **2017**, *7*, 1700666.
16. Y. Zhu, J. Dai, W. Zhou, Y. Zhong, H. Wang, Z. Shao, *J. Mater. Chem. A* **2018**, *6*, 13582.
17. Q. Sun, Z. Y. Dai, Z. B. Zhang, Z. L. Chen, H. Q. Lin, Y. Gao, D. J. Chen, *J. Power Sources* **2019**, *427*, 194.
18. X. Liu, L. Yang, Z. Zhou, L. Zeng, H. Liu, Y. Deng, J. Yu, C. Yang, W. Zhou, *Chem. Eng. J.* **2020**, *399*, 125779.
19. X. Xu, Y. Pan, Y. Zhong, L. Ge, S. P. Jiang, Z. Shao, *Composites Part B* **2020**, *198*, 108214.
20. B. He, K. Tan, Y. Gong, R. Wang, H. Wang, L. Zhao, *Nanoscale* **2020**, *12*, 9048.
21. H. Togano, K. Asai, S. Oda, H. Ikeno, S. Kawaguchi, K. Oka, K. Wada, S. Yagi, I. Yamada, *Mater. Chem. Front.* **2020**, *4*, 1519.
22. J. Gao, Y. Zhang, X. Wang, L. Jia, H. Jiang, M. Huang, A. Toghan, *Mater. Today Energy* **2021**, *20*, 100695.
23. D. Ji, C. Liu, Y. Yao, L. Luo, W. Wang, Z. Chen, *Nanoscale* **2021**, *13*, 9952.
24. C. Wu, Y. Sun, X. Wen, J.-Y. Zhang, L. Qiao, J. Cheng, K. H. L. Zhang, J*. Energy*

*Chem.* **2022**, *76*, 226.

1. S. Li, C. Cheng, A. Sagaltchik, P. Pachfule, C. Zhao, A. Thomas, *Adv. Funct. Mater.* **2019**, *29*, 1807419.
2. C. Wang, L. Zeng, W. Guo, C. Gong, J. Yang, *RSC Adv.* **2019**, *9*, 35646.
3. M. S. Alom, F. Ramezanipour, *ChemCatChem* **2021**, *13*, 3510.
4. Y. Bu, S. Kim, O. Kwon, Q. Zhong, G. Kim, *ChemElectroChem* **2019**, *6*, 1520.
5. R. K. Hona, S. B. Karki, T. Cao, R. Mishra, G. E. Sterbinsky, F. Ramezanipour, *ACS Catal.* **2021**, *11*, 14605.
6. Q. A. Islam, R. Majee, S. Bhattacharyya, *J. Mater. Chem. A* **2019**, *7*, 19453.
7. S. B. Karki, A. N. Andriotis, M. Menon, F. Ramezanipour, *ACS Appl. Energy Mater.* **2021**, *4*, 12063.
8. S. B. Karki, F. Ramezanipour, *ACS Appl. Energy Mater.* 2020, 3, 10983.
9. D. Guan, J. Zhou, Y.-C. Huang, C.-L. Dong, J.-Q. Wang, W. Zhou, Z. Shao, *Nat Commun* **2019**, *10*, 3755.
10. J. Dai, Y. Zhu, H. A. Tahini, Q. Lin, Y. Chen, D. Guan, C. Zhou, Z. Hu, H.-J. Lin, T.-S. Chan, C.-T. Chen, S. C. Smith, H. Wang, W. Zhou, Z. Shao, *Nat. Commun.* **2020**, *11*, 5657.
11. Y. Liu, Y. Dou, S. Li, T. Xia, Y. Xie, Y. Wang, W. Zhang, J. Wang, L. Huo, H. Zhao, *Small Methods* **2021**, *5*, 2000701.
12. J. Wang, Y. Gao, D. Chen, J. Liu, Z. Zhang, Z. Shao, F. Ciucci, *ACS Catal.* **2018**, *8*, 364.
13. Q. Chen, X. Han, Z. Xu, Q. Chen, Q. Wu, T. Zheng, P. Wang, Z. Wang, J. Wang, H. Li, *Nano Energy* **2023**, *110*, 108380.
14. Y. Wang, H. Yang, Z. Zhang, X. Meng, T. Cheng, G. Qin, S. Li, *J. Mater. Sci. Technol*. **2023**, *166*, 234.
15. D. Guan, H. Xu, Q. Zhang, Y.-C. Huang, C. Shi, Y.-C. Chang, X. Xu, J. Tang, Y. Gu, C.-W. Pao, S.-C. Haw, J.-M. Chen, Z. Hu, M. Ni, Z. Shao, *Adv. Mater.* **2023**, *35*, 2305074.
